# Supplementary material for: Splice-switching antisense oligonucleotide controlling tumor suppressor REST is a novel therapeutic medicine for neuroendocrine cancer
Source: Mol Ther Nucleic Acids. 2024 Jul 2;35(3):102250. doi: 10.1016/j.omtn.2024.102250 (PMC11456559; doi:10.1016/j.omtn.2024.102250)
Supplement: Document S1. Figures S1‒S8 and Tables S1‒S4 [file mmc1.pdf]

OMTN, Volume *35*

Supplemental information

Splice-switching antisense oligonucleotide controlling tumor suppressor REST is a novel therapeutic medicine for neuroendocrine cancer

Keishiro Mishima, Satoshi Obika, and Masahito Shimojo

Supplemental Information

Table S1. Sequences of the oligonucleotides

Name	Oligonucleotide sequence (5'→ 3')	Base	Used in
AmNA[+31/+48]	A(Y)^t^5(Y)^t^A(Y)^g^A(Y)^t^5(Y)^a^5(Y)^a^5(Y)^t^5(Y)^t^A(Y)^g	18	Figure 1<br>Figures S1&2
AmNA[-20/-3]	G(Y)^g^T(Y)^c^5(Y)^a^A(Y)^t^G(Y)^g^A(Y)~a^T(Y)^g^5(Y)^a^T(Y)^a	18	Figure 1<br>Figures S1&2
AmNA[+19/+36]	5(Y)^t^5(Y)^t^A(Y)^g^T(Y)^a^A(Y)^a^T(Y)^a^T(Y)^t^A(Y)^c^5(Y)^a	18	Figure 1<br>Figures S1&2
AmNA[+5/+22]	A(Y)^c^5(Y)^a^A(Y)^a^T(Y)^g^G(Y)^t^A(Y)^t^5(Y)^c^A(Y)^t^A(Y)^c	18	Figure 1<br>Figures S1&2
AmNA[-24/-7]	5(Y)^a^A(Y)^t^G(Y)^g^A(Y)^a^T(Y)^g^5(Y)^a^T(Y)^a^G(Y)^t^A(Y)^g	18	Figure 1<br>Figure S2

AmNA[-18/-1]	5(Y)^t^G(Y)^g^T(Y)^c^5(Y)^a^A(Y)^t^G(Y)^g^A(Y)^a^T(Y)^g^5(Y)^a	18	Figure 1<br>Figure S2
AmNA[+1/+18]	A(Y)^a^T(Y)^g^G(Y)^t^A(Y)^t^5(Y)^c^A(Y)^t^A(Y)^c^5(Y)^c^5(Y)^a	18	Figure 1<br>Figure S2
AmNA[+13/+30]	T(Y)^a^A(Y)^a^T(Y)^a^T(Y)^t^A(Y)^c^5(Y)^a^A(Y)^a^T(Y)^g^G(Y)^t	18	Figure 1<br>Figure S2
AmNA[+15/+32]	A(Y)^g^T(Y)^a^A(Y)^a^T(Y)^a^T(Y)^t^A(Y)^c^5(Y)^a^A(Y)^a^T(Y)^g	18	Figure 1<br>Figure S2
AmNA[+35/-2]	A(Y)^c^5(Y)^c^A(Y)^t^5(Y)^t^A(Y)^g^A(Y)^t^5(Y)^a^5(Y)^a^5(Y)^t	18	Figure 1<br>Figure S2
AmNA[+27/+44]	A(Y)^g^A(Y)^t^5(Y)^a^5(Y)^a^5(Y)^t^5(Y)^t^A(Y)^g^T(Y)^a^A(Y)^a	18	Figures 1&2<br>Figure S2
AmNA[+23/+40]	5(Y)^a^5(Y)^a^5(Y)^t^5(Y)^t^A(Y)^g^T(Y)^a^A(Y)^a^T(Y)^a^T(Y)^t	18	Figures 1&2

			Figure S2
AmNA[-44/-27]	A(Y)^a^A(Y)^c^G(Y)^g^A(Y)^a^A(Y)^t^T(Y)^g^A(Y)^c^A(Y)^t^T(Y)^t	18	Figure S1
AmNA[-32/-15]	T(Y)^g^5(Y)^a^T(Y)^a^G(Y)^t^A(Y)^g^A(Y)^a^A(Y)^a^A(Y)^c^G(Y)^g	18	Figure S1
AmNA[-17/-34]	G(Y)^c^A(Y)^t^A(Y)^a^G(Y)^a^G(Y)^t^A(Y)^a^T(Y)^a^5(Y)^a^T(Y)^t	18	Figure S1
AmNA[-8/+10]	5(Y)^c^A(Y)^t^A(Y)^c^5(Y)^c^5(Y)^a^5(Y)^t^G(Y)^g^T(Y)^c^5(Y)^a	18	Figure S1
AmNA[-5/-22]	T(Y)^a^5(Y)^a^T(Y)^t^5(Y)^t^A(Y)^c^5(Y)^t^G(Y)^a^A(Y)^t^A(Y)^c	18	Figure S1
AmNA[+43/-10]	G(Y)^a^A(Y)^t^A(Y)^c^A(Y)^t^A(Y)^c^5(Y)^t^G(Y)^a^A(Y)^t^A(Y)^c	18	Figure S1
AmNA[+23/+38]	5(Y)^a^5(Y)^t^5(Y)^t^A(Y)^g^T(Y)^a^A(Y)^a^T(Y)^a^T(Y)^t	16	Figure 2
AmNA[+25/+40]	5(Y)^a^5(Y)^a^5(Y)^t^5(Y)^t^A(Y)^g^T(Y)^a^A(Y)^a^T(Y)^a	16	Figure 2
AmNA[+27/+42]	A(Y)^t^5(Y)^a^5(Y)^a^5(Y)^t^5(Y)^t^A(Y)^g^T(Y)^a^A(Y)^a	16	Figure 2
AmNA[+29/+44]	A(Y)^g^A(Y)^t^5(Y)^a^5(Y)^a^5(Y)^t^5(Y)^t^A(Y)^g^T(Y)^a	16	Figure 2
AmNA[+26/+43]	G(Y)^a^T(Y)^c^A(Y)^c^A(Y)^c^T(Y)^c^T(Y)^a^G(Y)^t^A(Y)^a^A(Y)^t	18	Figure 2
AmNA[+27/+44]_1/3	A(Y)^g^a^T(Y)^c^a^5(Y)^a^c^T(Y)^c^t^A(Y)^g^t^A(Y)^a^a	18	Figure 2

AmNA[+25/+44]	A(Y)^g^A(Y)^t^5(Y)^a^5(Y)^a^5(Y)^t^5(Y)^t^A(Y)^g^T(Y)^a^A(Y)^a^T(Y)^a	20	Figure 2
AmNA[+27/+46]	5(Y)^t^A(Y)^g^A(Y)^t^5(Y)^a^5(Y)^a^5(Y)^t^5(Y)^t^A(Y)^g^T(Y)^a^A(Y)^a	20	Figure 2
AmNA[+23/+42]	A(Y)^t^5(Y)^a^5(Y)^a^5(Y)^t^5(Y)^t^A(Y)^g^T(Y)^a^A(Y)^a^T(Y)^a^T(Y)^t	20	Figure 2
AmNA[+21/+40]	5(Y)^a^5(Y)^a^5(Y)^t^5(Y)^t^A(Y)^g^T(Y)^a^A(Y)^a^T(Y)^a^T(Y)^t^A(Y)^c	20	Figure 2
AmNA[+23/+44]	A(Y)^g^A(Y)^t^5(Y)^a^5(Y)^a^5(Y)^t^5(Y)^t^A(Y)^g^T(Y)^a^A(Y)^a^T(Y)^a^T(Y)^t	22	Figure 2
NC1	5(Y)^a^5(Y)^c^T(Y)^a^T(Y)^c^G(Y)^t^A(Y)^a^A(Y)^c^A(Y)^c^T(Y)^c	18	Figure S3
NC2	5(Y)^c^A(Y)^c^5(Y)^t^A(Y)^g^5(Y)^a^A(Y)^t^A(Y)^t^5(Y)^a^5(Y)^t	18	Figure S3
NC3	5(Y)^g^5(Y)^c^A(Y)^c^A(Y)^a^5(Y)^t^A(Y)^t^5(Y)^a^5(Y)^t^A(Y)^t	18	Figure S3
NC4	5(Y)^a^5(Y)^g^A(Y)^a^A(Y)^t^5(Y)^t^A(Y)^t^5(Y)^c^5(Y)^c^A(Y)^t	18	Figure S3

The names, sequences, and base numbers of each oligonucleotide were shown. Each oligonucleotide was used in Figure 1, Figure 2, or Figures S1, 2, and 3.

5(Y)=AmNA_mC, N(Y)=AmNA, Lower case=DNA, ^Phosphorothioated.

Table S2. Higher-order structure prediction of REST_SSO by in silico analysis

Name	Oligonucleotide sequence (5'→ 3')	Intramolecular FE [kcal/mol]	Intermolecular FE [kcal/mol]
AmNA[+23/+42]	ATCACACTCTAGTAAATATT	−0.10	−1.05
AmNA[+21/+40]	CACACTCTAGTAAATATTAC	−0.23	−1.65
AmNA[+23/+38]	CACTCTAGTAAATATT	−0.03	−1.14
AmNA[+25/+40]	CACACTCTAGTAAATA	−0.09	−1.16
AmNA[+27/+46]	CTAGATCACACTCTAGTAAA	−3.49	−7.64
AmNA[+25/+44]	AGATCACACTCTAGTAAATA	−0.38	−2.13
AmNA[+27/+42]	ATCACACTCTAGTAAA	−0.09	−1.15
AmNA[+29/+44]	AGATCACACTCTAGTA	−0.37	−2.18
AmNA[+23/+44]	AGATCACACTCTAGTAAATATT	−0.38	−2.38
AmNA[+26/+43]	GATCACACTCTAGTAAAT	−0.14	−2.57
AmNA[-24/-7]	CAATGGAATGCATAGTAG	−0.61	−5.42
AmNA[-18/-1]	CTGGTCCAATGGAATGCA	−1.03	−9.17
AmNA[+1/+18]	AATGGTATCCATACCCCA	−2.48	−8.39
AmNA[+13/+30]	TAAATATTACCAAATGGT	−1.34	−5.61
AmNA[+15/+32]	AGTAAATATTACCAAATG	−0.38	−2.07
AmNA[+23/+40]	CACACTCTAGTAAATATT	−0.10	−1.09
AmNA[+27/+44]	AGATCACACTCTAGTAAA	−0.37	−2.08
AmNA[+35/-2]	ACCCATCTAGATCACACT	−0.01	−6.00

Free energy (FE) of the thermodynamic ensemble was calculated in a higher-order structure based on intramolecular or intermolecular interactions. Each sequence is shown without distinct AmNA and DNA. Refer to Table S1 for detailed sequences.

Table S3. Microarray analysis (mRNAs)

Gene Symbol	AmNA[+21/+40]		AmNA[+23/+44]		ChIP-Atlas (REST)
	Ratio	p-value	Ratio	p-value	Threshold (>500)
BPIFA4P	0.50	0.0489	0.90	0.0899	RE1
MIR762HG	2.46	<0.0001	2.08	<0.0001	(-)
KCNB1	0.48	0.0126	0.54	0.0695	RE1
ANKRD34C-AS1	0.38	0.0060	0.67	0.0452	RE1
XKR7	0.31	0.0005	0.74	0.0630	RE1
ZKSCAN8P1	3.02	0.0004	1.54	0.0199	(-)
SVOP	0.42	0.0004	0.83	0.0591	RE1
ACTL6B	0.45	0.0046	0.83	0.0138	RE1
KIAA0408	0.49	0.0078	0.84	0.1322	(-)
KEL	0.43	0.0311	0.77	0.4404	RE1
RRH	2.20	0.0009	1.29	0.0199	(-)
LAT2	4.98	0.0001	0.94	0.4110	(-)
CDO1	0.18	0.0334	0.33	0.1141	RE1
GABRD	0.45	0.0128	0.94	0.7998	RE1
DISP2	0.38	0.0068	0.86	0.0572	RE1
LOC107985773	0.48	0.0246	0.74	0.2462	(-)
SYN1	0.37	0.0009	0.75	0.0054	RE1
MIR7-3HG	0.21	0.0008	0.69	0.0201	RE1

CABP1	0.49	0.0132	0.95	0.7402	RE1
HRH3	0.47	0.0061	0.90	0.3754	RE1
SRRM3	0.44	0.0001	0.78	0.0046	RE1
UNC79	0.44	0.0011	0.91	0.2910	RE1
INSM2	0.40	0.0020	1.04	0.6396	RE1
SEZ6	0.29	0.0002	0.82	0.0298	RE1
CHGB	0.45	0.0008	0.81	0.0423	RE1
CPNE9	0.30	0.0031	0.74	0.1077	RE1
TMEM145	0.49	0.0036	0.82	0.1217	RE1
PSORS1C2	2.06	0.0477	1.50	0.2631	RE1
DNAI7	4.17	0.0100	2.44	0.0163	(-)
SPTBN4	0.41	0.0129	0.83	0.3644	RE1
C17orf98	4.98	0.0082	1.15	0.4590	(-)
CPLX2	0.29	0.0002	0.76	0.0367	RE1
KCNS2	0.33	0.0145	0.58	0.1056	RE1
UNC80	0.38	0.0331	0.62	0.1728	RE1
CDKN1A	2.97	0.0018	1.14	0.3836	RE1
GNAO1	0.32	0.0360	0.63	0.0109	RE1
SLITRK1	0.43	0.0037	0.88	0.2143	RE1
CACNA1B	0.42	0.0032	0.85	0.2236	RE1
SNTG1	0.49	0.0234	0.80	0.3731	RE1

KDSR	2.07	0.0047	1.18	0.0574	(-)
CHRNB2	0.25	0.0004	0.72	0.0114	RE1
TRIM67	0.32	0.0090	0.74	0.0243	RE1
KCNC1	0.38	0.0031	0.80	0.0741	RE1
HPCA	0.49	0.0132	0.81	0.1718	RE1
SCRT2	0.44	0.0003	0.88	0.1182	RE1
FCER1G	0.48	0.0277	0.64	0.1318	RE1
CHGA	0.43	0.0010	0.85	0.0688	RE1
RAB3C	0.44	0.0210	0.93	0.7265	RE1
SNAP25	0.37	0.0001	0.77	0.0169	RE1
RAB44	6.08	0.0007	1.01	0.9091	(-)
ATP1A1	0.33	0.0133	0.54	0.2141	RE1
MMP24	0.44	<0.0001	0.84	0.0407	RE1
LRIT3	15.97	0.0029	2.94	0.0133	(-)
SCRT1	0.42	0.0045	0.85	0.0290	RE1
GJA4	2.01	0.1412	3.11	0.0498	(-)
CALR3	1.18	0.3187	2.38	0.0006	(-)
PPFIA4	0.90	0.5339	0.48	0.0195	RE1
PRELID2	0.85	0.4259	0.43	0.0227	RE1
REST	1.14	0.1413	1.27	0.0855	RE1
SRRM4	0.77	0.0153	0.87	0.1940	RE1

Ratio compared with the negative control (NC) was shown based on the results of data analysis with changes of over 2-fold.

REST and SRRM4 were added to the list as reference RE1 genes.

Table S4. Microarray analysis (miRNAs)

Systematic name	AmNA[+21/+40]		AmNA[+23/+44]		AmNA7168	
	Ratio	p-value	Ratio	p-value	Ratio	p-value
hsa-miR-151a-3p	0.437	0.0407	0.612	0.1078	0.518	0.0616
hsa-miR-18b-5p	0.488	0.0444	0.598	0.0637	0.562	0.0774
hsa-miR-23b-3p	0.560	0.0566	0.640	0.1104	0.490	0.0286
hsa-miR-4281	1.917	0.0006	2.024	0.0006	1.982	0.0007
hsa-miR-4459	2.148	0.0148	2.225	0.0193	3.077	0.0012
hsa-miR-4465	1.404	0.0867	1.660	0.0535	2.020	0.0161
hsa-miR-4516	2.323	0.0026	2.793	0.0003	2.588	0.0010
hsa-miR-5703	3.879	0.0119	4.495	0.0069	5.545	0.0003
hsa-miR-5787	2.780	0.0007	3.225	0.0004	4.021	0.0000
hsa-miR-6087	1.704	0.0166	1.746	0.0232	2.040	0.0039
hsa-miR-6090	2.359	0.0000	2.528	0.0000	2.745	0.0001
hsa-miR-630	3.733	0.0097	4.215	0.0063	5.315	0.0004
hsa-miR-642a-3p	1.598	0.0128	1.795	0.0150	2.526	0.0005
hsa-miR-6858-3p	0.499	0.1212	0.541	0.2340	0.469	0.0474
hsa-miR-7107-5p	1.954	0.0053	1.794	0.0158	2.024	0.0011
hsa-miR-7150	3.624	0.0042	3.804	0.0031	5.227	0.0000
hsa-miR-762	2.107	0.0459	2.819	0.0120	2.844	0.0124
hsa-miR-8063	1.761	0.0012	1.943	0.0066	2.532	0.0002

hsa-miR-9-5p	0.430	0.0204	0.552	0.0505	0.559	0.0487

Ratio was analysed compared with the non-treatment control.

Figure S1.

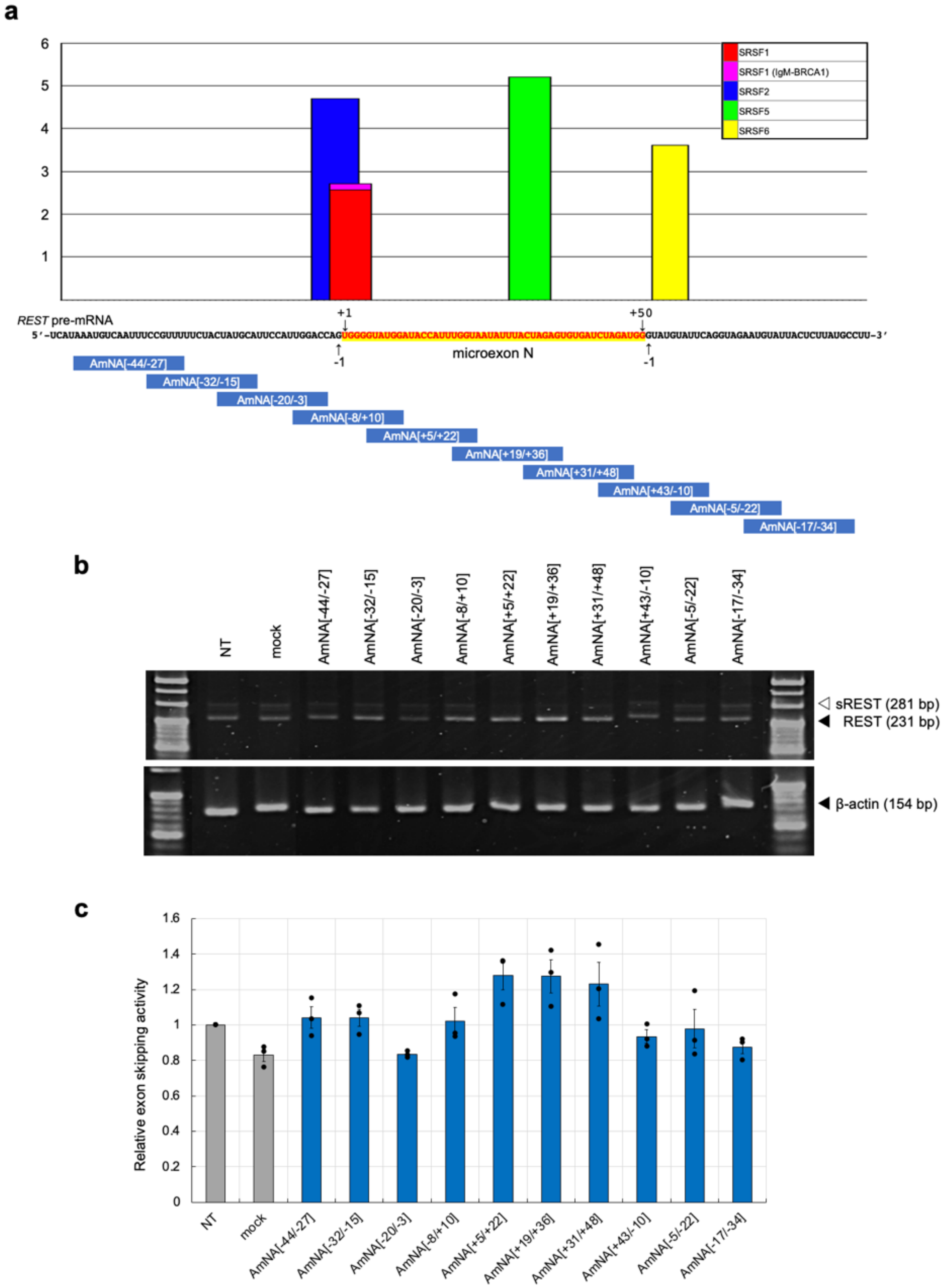
a
SRSF1
SRSF1 (IgM-BRCA1)
SRSF2
SRSF5
SRSF6
REST pre-mRNA
5′-UCAUAAAUGUCAAUUUCCGUUUUUCUACUAUGCAUUCCAUUGGACCAGUGGGGUAUGGAUACCAUUUGGUAAUAUUUACUAGAGUGUGAUCUAGAUGGGUAUGUAUUCAGGUAGAAUGUAUUACUCUUAUGCCUU-3′
+1
+50
-1
microexon N
-1
AmNA[-44/-27]
AmNA[-32/-15]
AmNA[-20/-3]
AmNA[-8/+10]
AmNA[+5/+22]
AmNA[+19/+36]
AmNA[+31/+48]
AmNA[+43/-10]
AmNA[-5/-22]
AmNA[-17/-34]
b
NT
mock
sREST (281 bp)
REST (231 bp)
β-actin (154 bp)
c
Relative exon skipping activity

Figure S1: Skipping of *REST* microexon N by REST_SSOs in STC-1 cells. a, Schematic of microexon N (highlighted in yellow) in the *REST* pre-mRNA. Oligonucleotides were designed around microexon N and are shown under *REST* pre-mRNA. The numbers in parenthesis indicate where the 5′ and 3′ ends are located on the pre-mRNA, with negative numbers indicating introns and positive numbers indicating exons. The diagram above the *REST* pre-mRNA shows the prediction of the SRSF-binding positions and ESE homology score on the vertical axis based on ESEfinder3.0. **b,** Screening of REST SSOs using SCLC cells (STC-1). STC-1 cells were transfected with each oligonucleotide (final concentration, 10 nM) via electroporation. After 24 h of transfection, RNA was extracted, and the REST band was analysed via agarose gel electrophoresis. **c,** Exon skipping activity for each oligonucleotide was evaluated compared with that of NT, which was set as 1 (n = 3, mean ± standard error of mean). Exon skipping activity was defined as the intensity of the REST band relative to the sum of the intensities of the REST and sREST bands. NT: non-treatment; mock: lipofection without SSO.

Figure S2

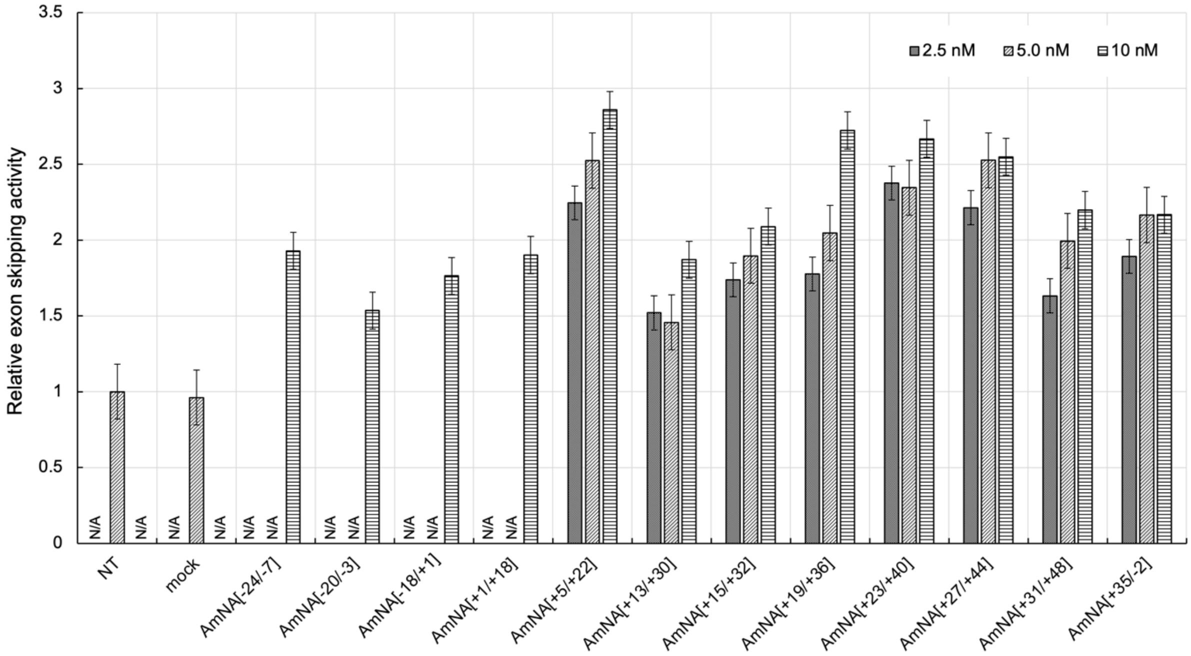

Figure S2: Analysis of exon skipping by REST SSOs in VCaP cells. The PCa cell line VCaP was transfected with various amounts of oligonucleotides, and total RNA was extracted after 48 h. RT-PCR was performed to assess *REST* splicing, and exon skipping activity was analysed based on band intensities on polyacrylamide gels. Exon skipping activity is shown compared with that of NT, which was set as 1 (n = 3, mean ± standard error of mean). NT: non-treatment; mock: lipofection without SSO. AmNA[-24/-7], AmNA[-20/-3], AmNA[-18/+1], and AmNA[+1/+18] were used at a concentration of 10 nM, whereas NT and mock were used at 5.0 nM. NA; not available.

Figure S3

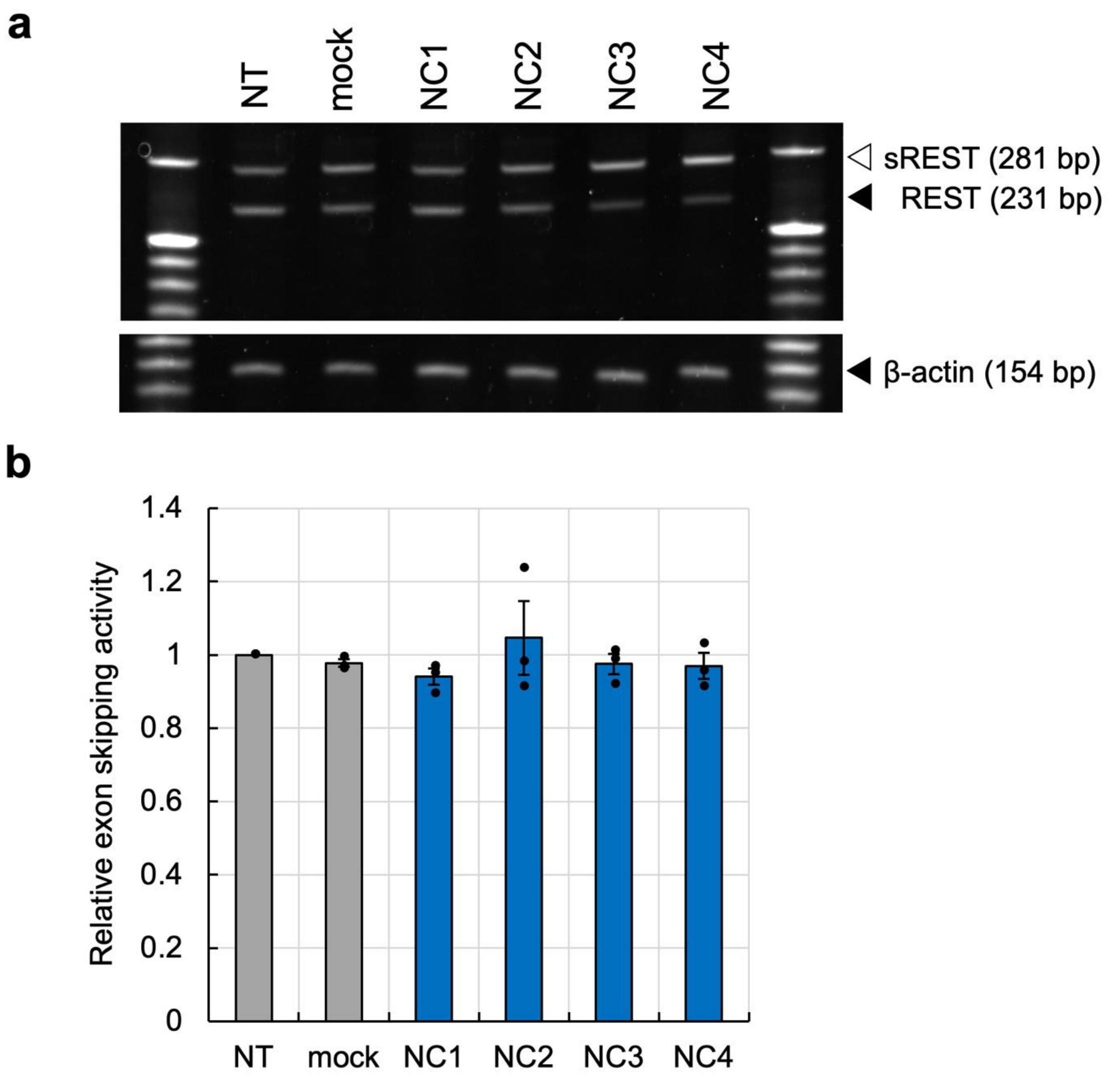

Figure S3: Assessment of negative control oligonucleotides using the PCa cell line VCaP. **a,** VCaP cells were transfected with each oligonucleotide (NC1-4) (final concentration: 10 nM) using Lipofectamine, and total RNA was extracted after 24 h. RT-PCR was performed and REST (231 bp) and sREST (281 bp) were separated via polyacrylamide gel electrophoresis. β-actin (154 bp) was used as the internal standard. **b,** Exon skipping activity for each oligonucleotide was evaluated compared with that of the NT control, which was set as 1 (n = 3, mean ± standard error of mean). Exon skipping activity was defined as the intensity of the REST band relative to the sum of the intensities of the REST and sREST bands. NT: non-treatment; mock: lipofection without SSO.

Figure S4

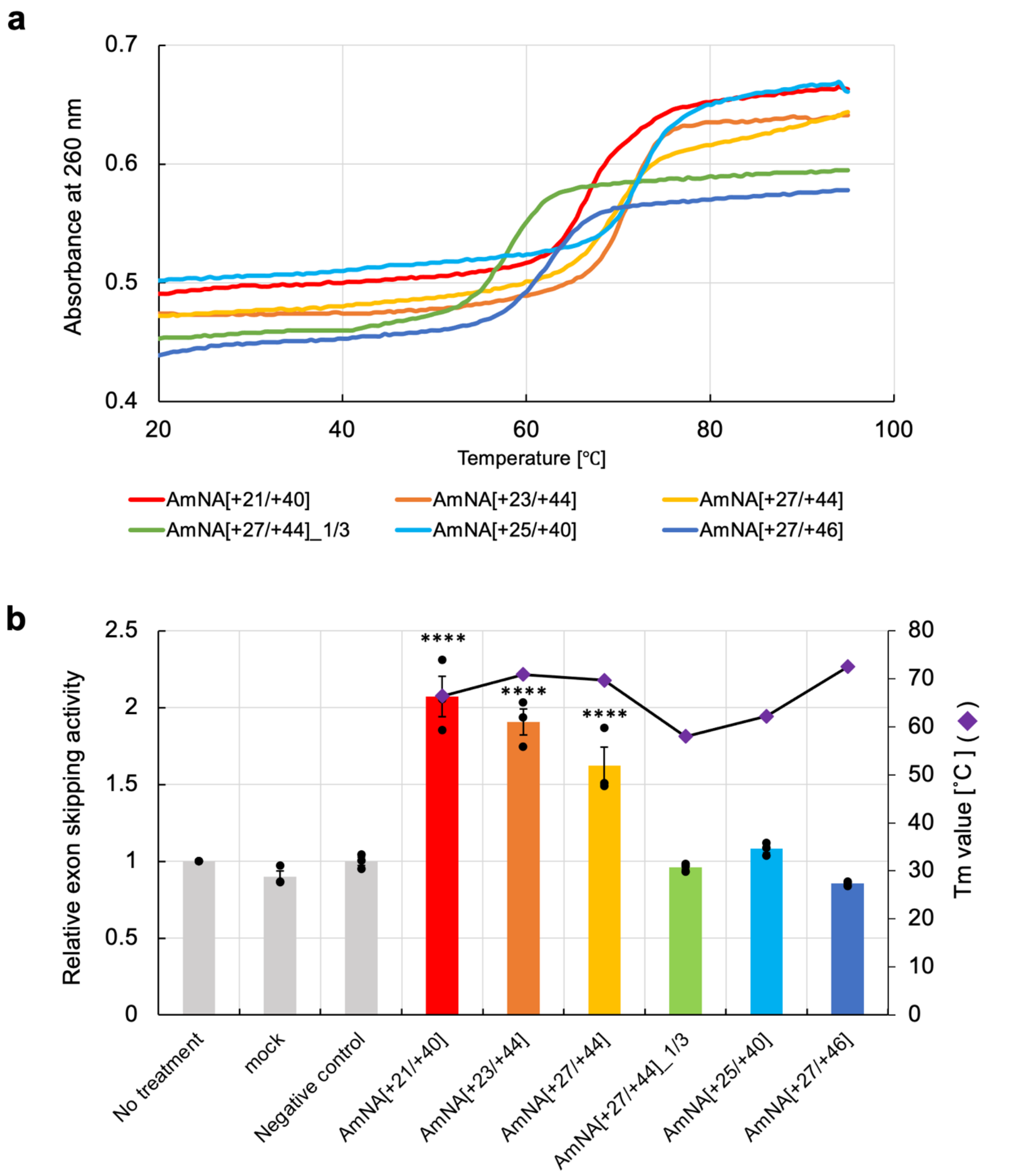

Figure S4: *T*m evaluation of each oligonucleotide. a, *T*m melting curves for each oligonucleotide. **b,** Each oligonucleotide was analysed based on the melting temperature curve. Exon skipping activity is shown in the bar graph, and *T*m indicated on the right is shown as a diamond above the bar graph. mock: lipofection without SSO. Statistical significance is expressed in comparison with values for no treatment and was analysed using one-way analysis of variance (ANOVA) followed by Dunnett's t-test. ****P < 0.0001.

Figure S5

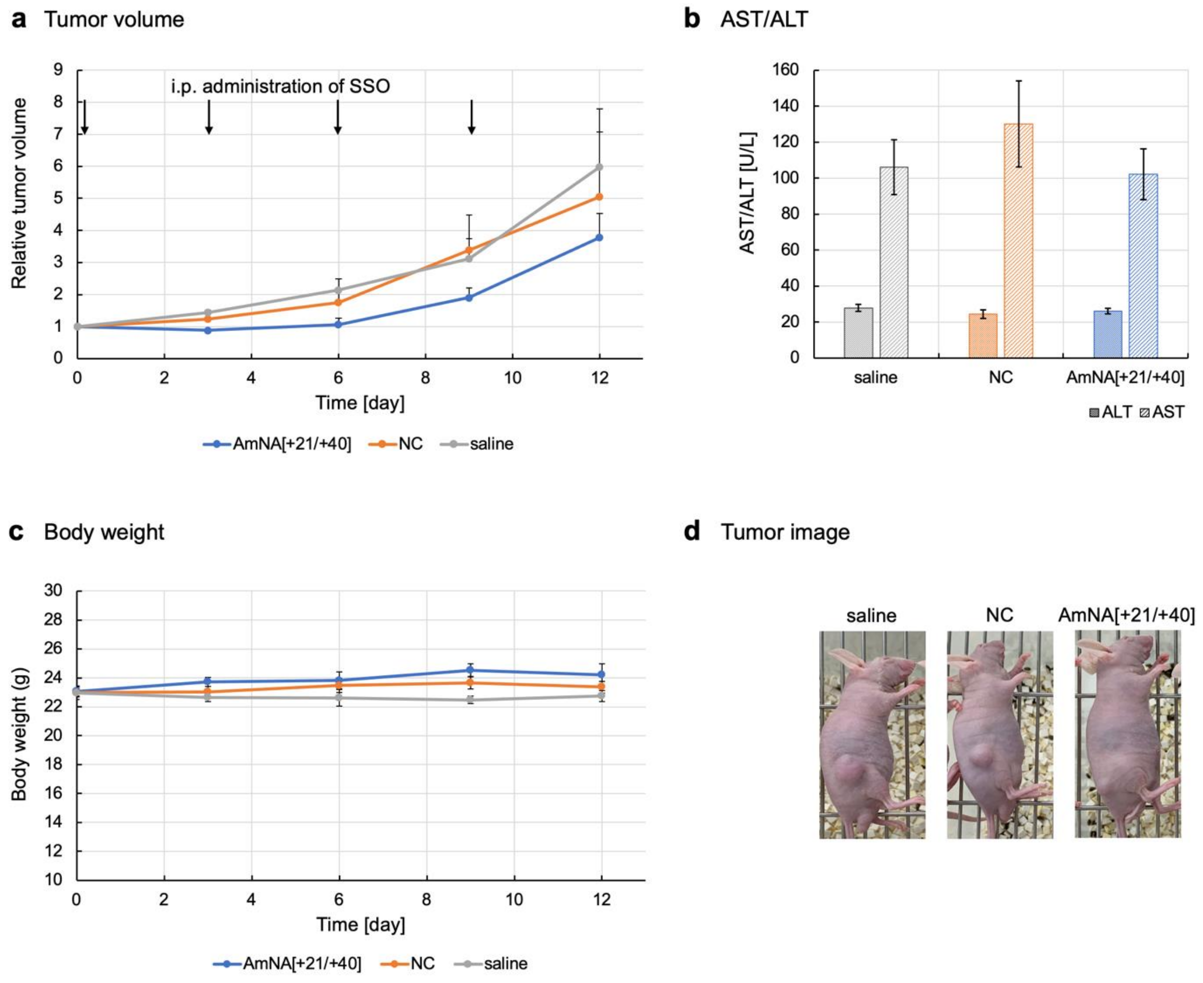

Figure S5: Antitumour effects after AmNA[+21/+40] administration in xenograft mice bearing tumours derived from SCLC cell line N417. a, Antitumour effects upon AmNA[+21/+40] administration in SCLC tumour-bearing xenograft mice. N417 cells (5.0 $\times 10^5$) were subcutaneously implanted into 8-week-old BALB/c Slc-nu/nu mice (n = 5); after 7 days, saline or oligonucleotides (AmNA[+21/+40] or NC) were intraperitoneally administered at 10 mg/kg every 3 days. Tumour volume was calculated based on the long and short diameters. **b,** AST/ALT tests using blood samples. Blood samples were collected on day 12 and used for the ALT/AST assay. **c**, Body weight was measured every 3 days. **d,** Images of tumours observed for mice from different groups. NC; negative control.

Figure S6

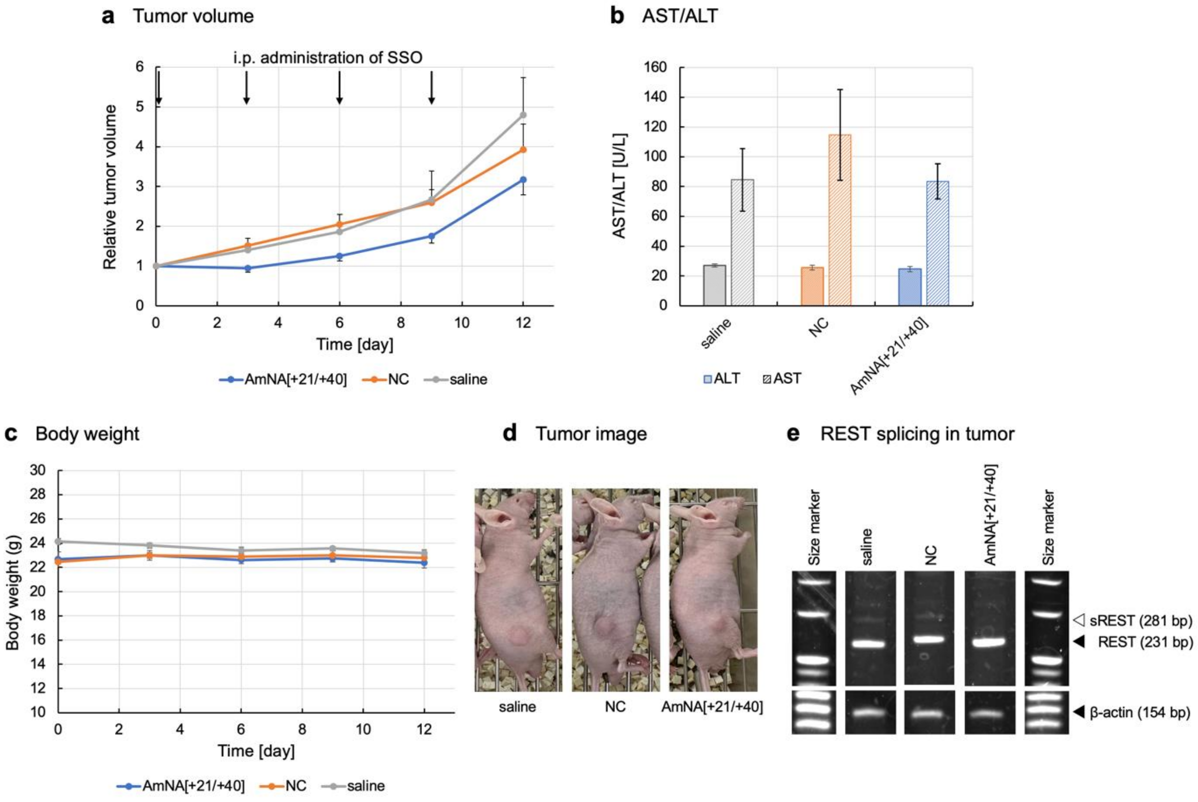

Figure S6: Antitumour effects upon AmNA[+21/+40] administration in xenograft mice bearing tumours derived from the PCa cell line 22Rv1. a, Antitumour effects upon AmNA[+21/+40] administration in PCa tumour-bearing xenograft mice. 22Rv1 cells (5.0×10^5) were subcutaneously implanted into 8-week-old BALB/c Slc-nu/nu mice (n = 5); after 7 days, saline or oligonucleotides (AmNA[+21/+40] or NC) were intraperitoneally administered at a dose of 10 mg/kg every 3 days. Tumour volume was calculated. **b,** AST/ALT test using blood samples on day 12. **c**, Body weight was measured every 3 days. **d,** Images of tumours observed for mice from different groups. **e,** *REST* splicing in the tumour was analysed. NC; negative control.

Figure S7

a Standard curve

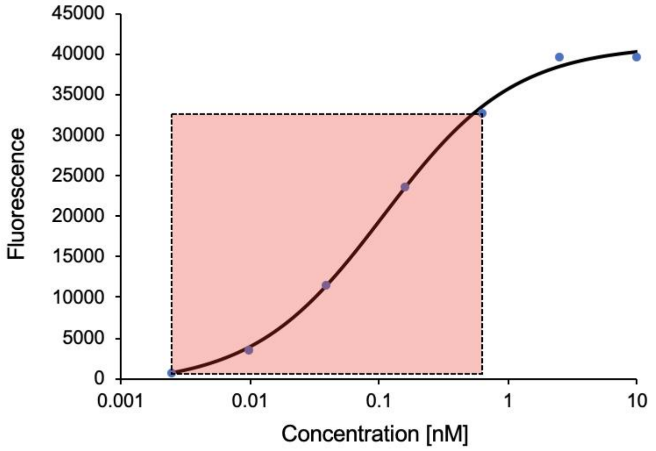

b Principle of ELOSA

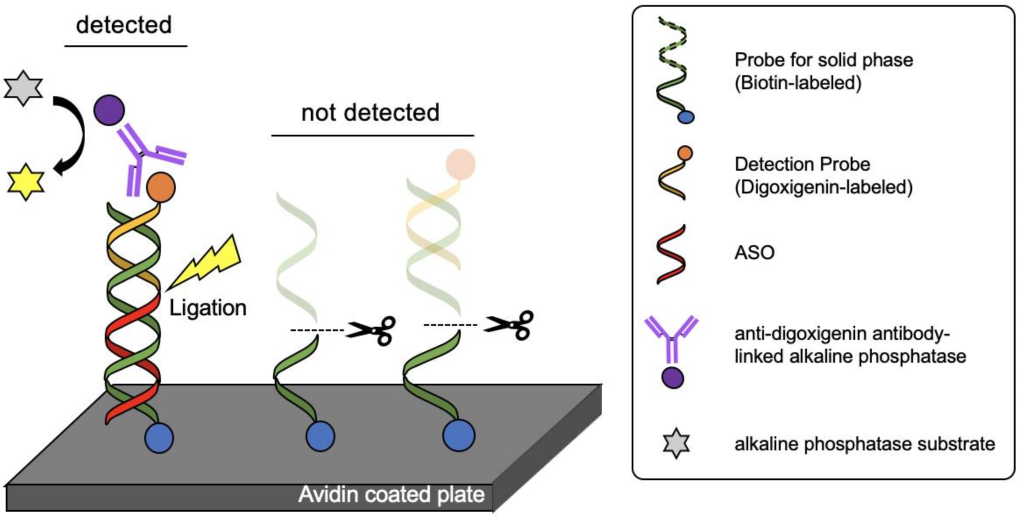

Figure S7: Principle of oligonucleotide quantification by enzyme-linked oligosorbent assay (ELOSA). a, Standard curve obtained using known amounts of AmNA[+21/+40]. **b,** Principle of ELOSA.

Figure S8.

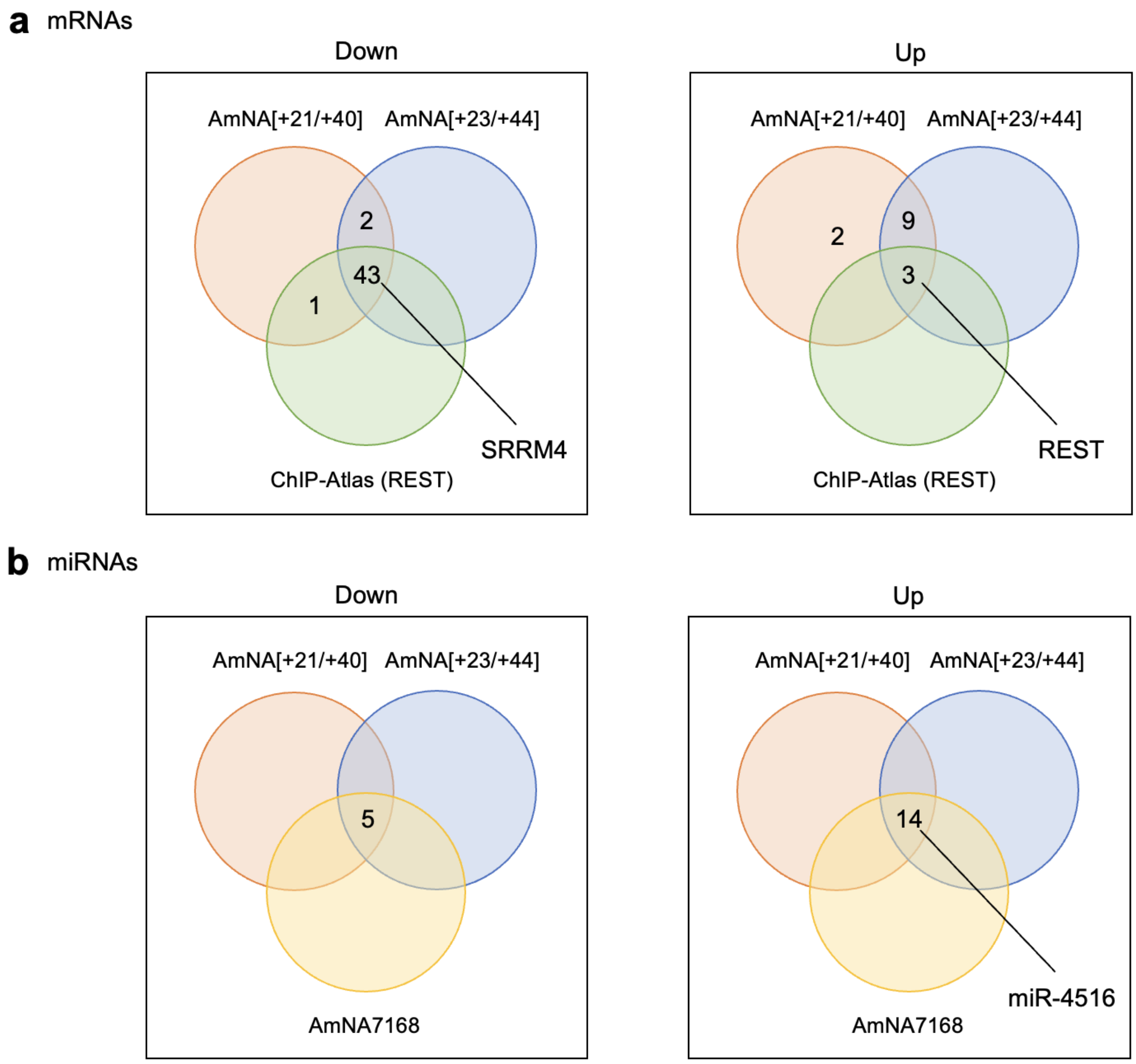

Figure S8: Microarray analysis in 22Rv1 cell transfected with AmNA[+21/+40] or AmNA[+23/+44]. The number of genes whose expression levels changed by more than 2-fold has been shown in the Venn diagram. **a,** mRNA **b,** miRNA
