# Supplementary material for: Splice-switching antisense oligonucleotide controlling tumor suppressor REST is a novel therapeutic medicine for neuroendocrine cancer
Source: Mol Ther Nucleic Acids. 2024 Jul 2;35(3):102250. doi: 10.1016/j.omtn.2024.102250 (PMC11456559; doi:10.1016/j.omtn.2024.102250)
Supplement: Document S2. Article plus supplemental information [file mmc2.pdf]

Molecular Therapy
Nucleic Acids
Original Article

Splice-switching antisense oligonucleotide controlling tumor suppressor REST is a novel therapeutic medicine for neuroendocrine cancer

Keishiro Mishima,[1] Satoshi Obika,[1,2,3] and Masahito Shimojo[1]

[1]Graduate School of Pharmaceutical Sciences, Osaka University, Osaka 565-0871, Japan; [2]Institute for Open and Transdisciplinary Research Initiatives (OTRI), Osaka University, Osaka 565-0871, Japan; [3]National Institutes of Biomedical Innovation, Health, and Nutrition (NIBIOHN), Osaka 567-0085, Japan

RNA splicing regulation has revolutionized the treatment of challenging diseases. Neuroendocrine cancers, including small cell lung cancer (SCLC) and neuroendocrine prostate cancer (PCa), are highly aggressive, with metastatic neuroendocrine phenotypes, leading to poor patient outcomes. We investigated amido-bridged nucleic acid (AmNA)-based splice-switching oligonucleotides (SSOs) targeting RE1-silencing transcription factor (REST) splicing as a novel therapy. We designed AmNA-based SSOs to alter REST splicing. Tumor xenografts were generated by subcutaneously implanting SCLC or PCa cells into mice. SSOs or saline were intraperitoneally administered and tumor growth was monitored. Blood samples were collected from mice after SSO administration, and serum alanine aminotransferase and aspartate aminotransferase levels were measured to assess hepatotoxicity using a biochemical analyser. *In vitro*, REST_SSO reduced cancer cell viability. In a tumor xenograft model, it exhibited significant antitumor effects. It repressed REST-controlled RE1-harboring genes and upregulated miR-4516, an SCLC biomarker. Our findings suggest that REST_SSO suppresses tumorigenesis in neuroendocrine cancers by restoring REST function. This novel therapeutic approach holds promise for intractable neuroendocrine cancers such as SCLC and neuroendocrine PCa.

INTRODUCTION

Neuroendocrine (NE) cancer is an aggressive, poorly differentiated, and high-grade tumor.[1] Small cell lung cancer (SCLC) and prostate cancer (PCa) with NE differentiation (NEPCa) are characterized by the NE phenotype, high invasiveness, and metastatic potential. Currently, the therapeutic outcomes of SCLC remain poor, and effective therapies are urgently needed. Androgen deprivation therapy is effective for some patients with PCa; however, most patients may develop castration resistance, with the development of highly metastatic PCa and eventually NEPCa. NEPCa is the most aggressive form of PCa, with no available treatment.[2] NE phenotypes in SCLC and PCa are regulated by repressing RE1 *cis* element-containing genes via RE1-silencing transcription factor (REST).[3] REST is an oncoprotein that was identified via systematic analysis.[4] In aggressive NE cancers, such as SCLC or PCa, the mRNAs of splicing isoforms of *REST* (sREST)[5] and serine/arginine repetitive matrix 4 (*SRRM4*) are abnormally highly expressed.[6,7] SRRM4 (nSR100) is a splicing activator[8] specifically expressed in the normal brain tissues; it induces the splicing of *REST* mRNA into *sREST* mRNA. The aberrant expression of *sREST* mRNA, but not of full-length *REST* mRNA, is a hallmark of the NE phenotypes of SCLC and PCa and possibly of aggressive breast cancer types.[9] The expression of *sREST* mRNA owing to *REST* splicing changes upon the insertion of microexon N into the *REST* mRNA. This incorporation produces a loss-of-function REST,[10] possibly caused by nonsense-mediated mRNA decay (NMD).[11,12] Regulation of *REST* splicing is involved in many biological functions, and its disruption can cause various diseases,[13] including NE phenotype in tumors.[14] Abnormal splicing changes the expression ratio of *sREST* mRNA to *REST* mRNA, resulting in the re-expression of RE1 genes, leading to abnormal differentiation and proliferation. Thus, *REST* splicing regulation is a target for developing new treatment alternatives for many diseases.[15,16]

We previously developed an antisense oligonucleotide (ASO) targeting *SRRM4*; it is a gapmer structure that contains a central block of DNA with a wing region of artificial amido-bridged nucleic acids (AmNAs) and exhibits high affinity for its target *SRRM4* mRNA.[17] AmNA is an artificial nucleic acid[18] that was newly synthesized by our group. The single-stranded SRRM4_ASO specifically binds to target *SRRM4* mRNA sequences and induces mRNA degradation by RNase H.[17,19] SRRM4_ASO induces antitumor effects by switching *REST* splicing from *sREST* mRNA.[20] Therefore, ASO is an effective treatment option for intractable diseases.[21,22] The splice-switching oligonucleotide (SSO) is also an ASO that mediates the blocking of the serine/arginine-rich splicing factor (SRSF)-binding site in an exonic splicing enhancer.[23,24] The structure of SSO differs from that of ASO, which prevents RNase H activation; furthermore, artificial nucleic acid and DNA are alternatively complexed in the structure, known as a mixmer SSO. Since 2022, five SSO-based medicines have been approved for treating spinal muscular atrophy and

Received 25 February 2024; accepted 10 June 2024; Published: July 2, 2024.
https://doi.org/10.1016/j.omtn.2024.102250.

Correspondence: Masahito Shimojo, Graduate School of Pharmaceutical Sciences, Osaka University, Osaka 565-0871, Japan.
E-mail: shimojo@phs.osaka-u.ac.jp

© 2024 The Authors.
Published by Elsevier Inc. on behalf of The American Society of Gene and Cell Therapy.
This is an open access article under the CC BY license (http://creativecommons.org/licenses/by/4.0/).

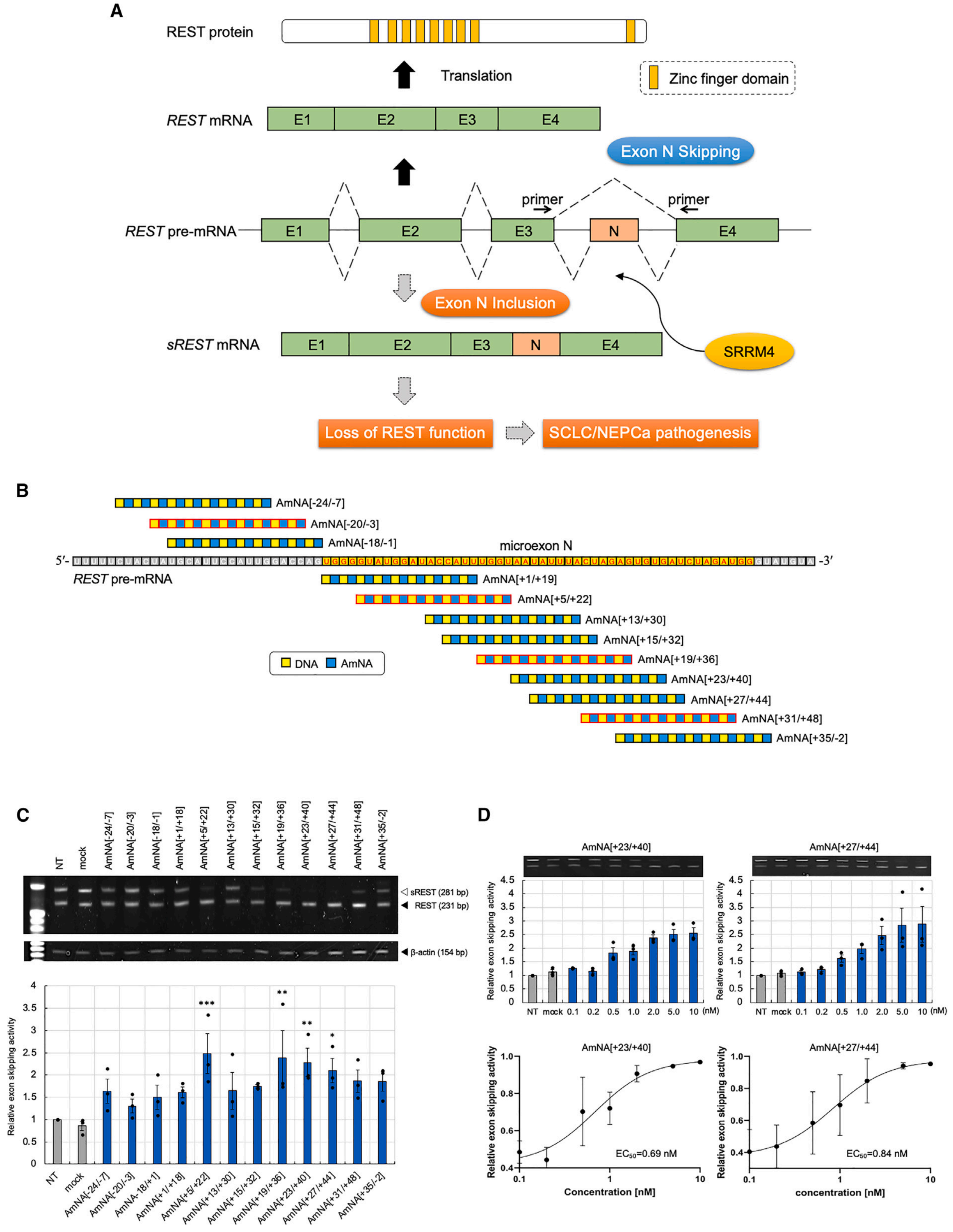

(legend on next page)

Duchenne muscular dystrophy.[25] However, SSO-based medicines for cancer treatment have not yet been approved by the Food and Drug Administration. Appropriate splicing changes are important for gene regulation during cancer therapy.[26–28] Here, we developed REST_SSO, which directly regulates *REST* splicing (Figure 1A), to overcome the dysregulation of REST to achieve antitumour effects.

RESULTS

Screening of REST_SSO

We constructed 18-mer ASOs around *REST* microexon N and synthesized them for the first-level screening (Table S1). SSOs are single-stranded oligonucleotides that can easily adopt many secondary structures, including loops and self-dimerized forms. A higher-order secondary structure greatly reduces SSO binding to the target sequence, leading to a decrease in activity.[29] *In silico* secondary structure prediction (RNAfold) was performed to avoid the loss of exon skipping activity due to secondary structure formation (Table S2). The free energy (FE) of the dynamic assemblies was calculated, and a stable higher-order structure was predicted to be below zero for FE. SSOs are mixmers that contain AmNA and DNA, and all nucleotides are linked by a phosphorothioate linkage that prevents nuclease-mediated degradation.[30] Artificial nucleic acids, including AmNAs, contribute toward an improved affinity for target sequences, nuclease resistance, and low toxicity.[18,31] The introduction of AmNA in SSO resulted in higher exon skipping activity than that observed for locked nucleic acid-modified SSO.[29] For the initial screening, *REST* exon skipping activity has been illustrated in a graph depicting the prediction of SRSFs around microexon N (Figure S1). SSOs were further designed based on AmNA[−20/−3], AmNA[+5/+22], AmNA[+19/+36], and AmNA [+31/+48] (Figure 1B), and exon skipping activity was analyzed (Figure 1C) for dose dependency (Figure S2). We initially screened SSOs in STC1 cells using electroporation for higher transfection efficiency but showed slight cell toxicity. Thus, we switched to Lipofectamine 3000, which showed higher transection with much less toxicity, and analyzed SSO dose dependency in VCaP cells (Figure S2). VCaP cells, a human PCa cell line, were transfected with each SSO using Lipofectamine 3000, and *REST* and *sREST* mRNA expression were measured (Figure 1C). Forward and reverse primers shown were used to amplify between exons 3 and 4 (Figure 1A). The band corresponding to *sREST* mRNA containing microexon N was 281 bp, whereas that corresponding to *REST* mRNA without microexon N was 231 bp. Each band was excised from the gel and confirmed by Sanger sequencing. Exon skipping activities were calculated as the intensity of the lower band (*REST* mRNA) relative to the total intensities of the upper (*sREST* mRNA) and lower (*REST* mRNA) bands. Exon skipping activity mainly depended on SSO concentrations. AmNA[+23/+40] and AmNA[+27/+44] exhibited higher exon skipping activity (Figure S2). The half-maximal effective concentration (EC_{50}) of each SSO was analyzed using concentration dependency; AmNA[+23/+40] and AmNA[+27/+44] exhibited relatively lower EC_{50} values of 0.69 and 0.84 nM, respectively (Figure 1D).

To further optimize the SSO sequence for higher exon skipping activity, we constructed oligonucleotides based on AmNA[+23/+40] and AmNA[+27/+44] (Figure 2A). Each SSO was transfected into VCaP cells, followed by the assessment of exon skipping activity in comparison with that observed for the non-treatment (NT) control. AmNA[+26/+43] was constructed as an alternative replacement between DNA and AmNA compared with AmNA[+27/+44]; however, the sequence was shifted by one base, owing to the limitation of SSO synthesis containing AmNA. As a result of exon skipping analysis (Figures 2B and 2C), AmNA[+21/+40] exhibited the highest exon skipping activity and was selected for further analysis. Although we used NT cells and mock (transfection reagent only) for the initial screening, the negative control (NC) was selected based on an 18-mer oligonucleotide scrambled sequence based on AmNA[+35/−2] that did not show complementary binding, even in the case of one base mismatch, as assessed by the GGGenome Database (https://gggenome.dbcls.jp/en/) and REST exon skipping analysis using VCaP cells (Figure S3). The scrambled oligonucleotides were screened using VCaP cells, all of which were negative in terms of REST splicing in 22Rv1, as well as in several other cell lines, N417 and H146 cells (see “NC” in Figures 2 and 3). Based on our analysis, NC3 was used as a NC oligonucleotide in this study.

REST_SSO regulates REST splicing in NEPCa and SCLC cells

AmNA[+21/+40] and AmNA[+23/+44] were transfected into another PCa cell line, 22Rv1, and two SCLC cell lines, H146 and N417, followed by exon skipping analysis. All NE cancer cell lines abnormally express *sREST* mRNA,[20] which is produced via aberrant REST splicing by SRRM4.[6] To compare cell viability in the three cell lines, we optimized transfection and used the same lipofection method. In all the tested cell lines, AmNA[+21/+40] and AmNA [+23/+40] showed REST splicing activity, while AmNA[+21/+40]

Figure 1. Development of AmNA-based REST_SSOs around microexon N on *REST* pre-mRNA

(A) Splicing of *REST* is regulated by SRRM4-mediated microexon N insertion. *REST* microexon N (shown in red) is inserted into pre-mRNA between E3 and E4 by SRRM4, thereby lowering functional REST expression possibly via NMD. REST contains 9 zinc finger domains (shown in orange) that are important for its proper function. REST_SSO induces microexon N insertion skipping by interfering with SRRM4 binding, thereby upregulating functional REST expression. (B) SSO candidate oligonucleotides were designed around microexon N nucleotides (red characters shown in yellow boxes). All 18-mer SSOs contain phosphorothioate backbone linkages, with alternating AmNA and DNA as a mixmer SSO. These modifications contribute to improved affinity toward the target sequence, nuclease resistance, and low toxicity. Each oligonucleotide in the scheme shows the position below *REST* pre-mRNA. (C) After transfecting each oligonucleotide (10 nM) in VCaP cells, total RNA was extracted, and RT-PCR was performed using REST-specific primers, followed by polyacrylamide gel analysis. *REST* microexon N skipping activities of oligonucleotides are shown based on the band intensities. (D) The dose dependency of *REST* exon skipping activity by AmNA[+23/+40] and AmNA[+27/+44] was assayed, and EC_{50} was determined. Significance was analyzed as compared with the NT control using 1-way ANOVA followed by Dunnett’s t test. **p* < 0.05; ***p* < 0.01; ****p* < 0.001.

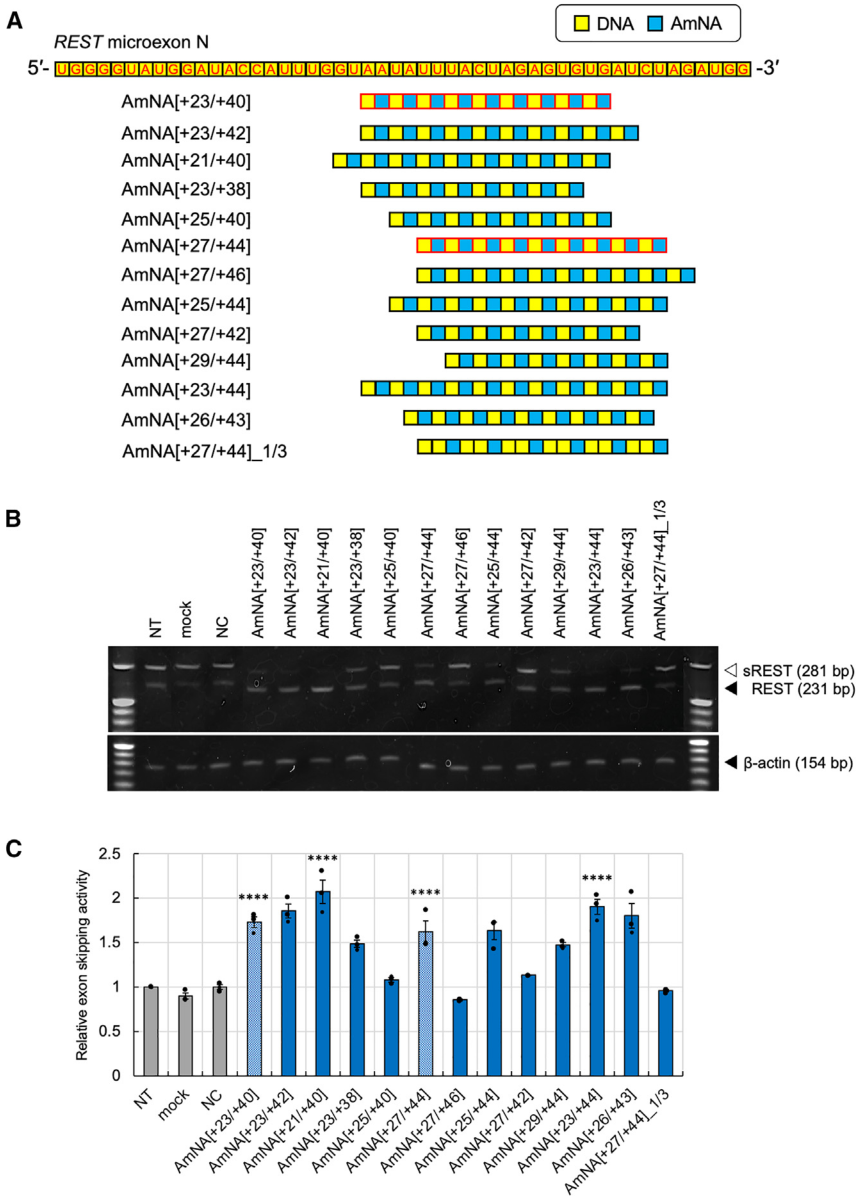

Figure 2. Optimization of REST_SSOs in VCaP, a PCa cell line

(A) Schematic of the designed REST_SSOs based on AmNA[+23/+40] and AmNA[+27/+44] under *REST* microexon N sequence. (B) The PCa cell line, VCaP, was transfected with each oligonucleotide (10 nM final concentration) using lipofection. Total RNA was extracted from the collected cells 48 h later, and RT-PCR and native PAGE were performed. (C) Exon skipping activity of each oligonucleotide was evaluated compared with that in the NT control, which was set as 1 (n = 3, mean ± SEM). Statistical significance was analyzed as compared with the values for NT using 1-way ANOVA followed by Dunnett's t test. ****p < 0.0001.

induced REST splicing slightly more effective than AmNA[+23/+44] (Figure 3). The exon skipping activity of AmNA[+21/+40] and AmNA[+23/+44] relatively correlated with their melting temperature (T_m) and FE values. However, AmNA[+27/+46] exhibited considerably lower exon skipping activity—even with higher T_m values—than AmNA[+21/+40] and AmNA[+23/+44], presumably owing to the self-secondary conformation[32] and corresponding FE values (Figure S4; Table S2).

SCLC and PCa cell viability is affected by REST_SSO AmNA [+21/+40] and AmNA[+23/+44]

Cell viability was analyzed using REST_SSO. After optimizing the oligonucleotide concentration for cell viability analysis up to 72 h, we selected 50 nM oligonucleotides (for 22Rv1) or 100 nM oligonucleotidse (for VCaP, N417, and H146). Cells were transfected with AmNA[+21/+40] using lipofection, and cell viability was quantified every 24 h for 72 h (Figure 4). We used the gapmer SRRM4_ASO targeting *SRRM4*[17,20] as the positive control for the cell viability assay. NCs and mock control (Lipofectamine only) showed that the relative cell viability increased until 72 h, whereas REST_SSO (AmNA[+21/+40]) and SRRM4_ASO considerably suppressed the viability. Viability 72 h post-transfection is shown in the bar graph (Figure 4, right). Compared with the NT of PCa (22Rv1 and VCaP) (Figures 4A and 4B) and SCLC (NCI-N417 and NCI-H146) (Figures 4C and 4D) cell lines, REST_SSO (AmNA[+21/+40]) and SRRM4_ASO considerably decreased the cell viability. Although SRRM4_ASO exerted much stronger effects than REST_SSO, particularly in PCa, this may have been because of a time lag in abnormal SRRM4 suppression, either indirectly by REST_SSO or directly by SRRM4_ASO. Another reason may be that SCLC is a highly heterogeneous cancer.[33,34] We confirmed that the SCLC cell lines used in our study contain stem cell-like populations (data not shown).

REST_SSO exerts antitumor effects in an NE cancer xenograft mouse model

To analyze the antitumor effects of AmNA[+21/+40], we used xenograft mice transplanted with human cancer cells. SCLC cells were transplanted into xenograft mice. AmNA[+21/+40] was then intraperitoneally administered 4 times at a dose of 10 mg/kg every 3 days, and the tumor volume was analyzed (Figure S5). The tumor volume reduced compared with that in the NC or saline control; however, the difference was not statistically significant. This may be due to the duration of AmNA[+21/+40] administration or the

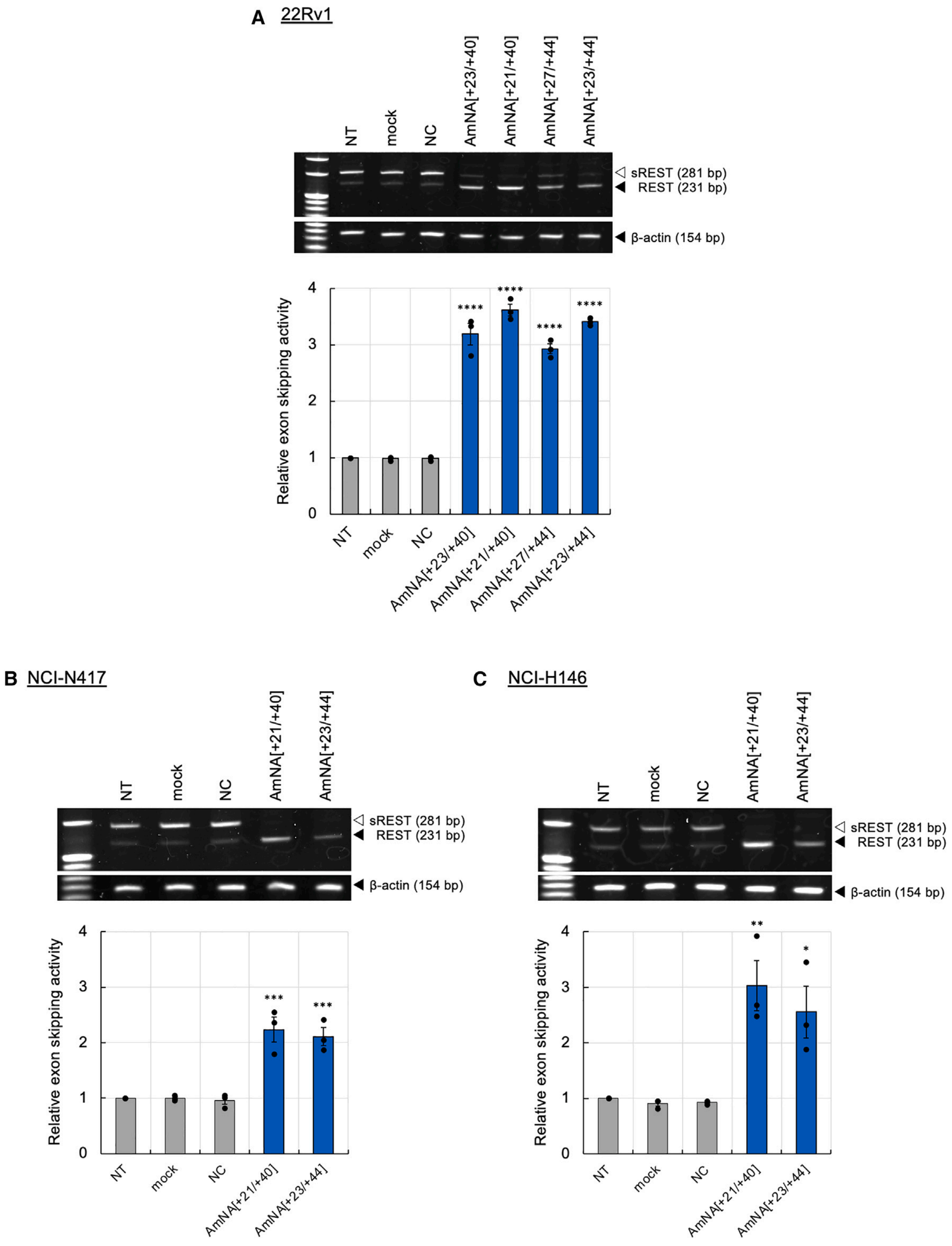

Figure 3. *REST* exon skipping in PCa and SCLC cells

(A) Exon skipping activity of REST_SSO was analyzed in the PCa cell line 22Rv1. 22Rv1 cells (0.1×10^6) were transfected with 10 nM of each SSO using lipofection and cultured for 48 h. After total RNA extraction, RNA (100 ng) was amplified via RT-PCR, followed by PAGE. (B and C) The SCLC cell line NCI-N417

(legend continued on next page)

high heterogeneity of cancer stem cells in the tumor. We then used the PCa cell line 22Rv1 to construct a xenograft mouse model, followed by the intraperitoneal administration of AmNA[+21/+40] 4 times at a dose of 10 mg/kg every 3 days, and then analyzed the tumor volume. AmNA[+21/+40] administration tended to substantially reduce the tumor size. However, the tumor size did not statistically change (Figures S6A and S6D), and the body weight or plasma alanine aminotransferase/aspartate aminotransferase (AST/ALT) levels in mice were not affected (Figures S6B and S6C). *REST* splicing analysis revealed that *sREST* mRNA was decreased in tumors compared with NC or saline control; however, *REST* mRNA was highly detected in all groups owing to the inclusion of mouse *REST* mRNA (Figure S6E); we obtained results similar to those demonstrated in Figure S5. Intratumoral *REST* splicing analysis by conventional RT-PCR separating human and mouse *REST* was difficult owing to the high homology of microexon N between human and mouse *REST* sequences. AmNA[+21/+40] administration did not affect the body weight or plasma AST/ALT levels in mice. We hypothesized that the antitumor effect was affected by the lower uptake of AmNA[+21/+40] into tumor cells and constructed cyclic arginine/glycine/aspartic acid (cRGD)-conjugated AmNA [+21/+40] to facilitate cellular uptake. cRGD, such as that used in cRGD-conjugated microRNAs (miRNAs), is used to induce cellular uptake by interacting with integrin.[35,36] To increase the effect of REST_SSO in xenograft mice, we intraperitoneally administered cRGD-conjugated AmNA[+21/+40] under the above-mentioned conditions, except that the dose was administered every 2 days, to increase the amount of SSO in the body; thereafter, tumor volume was analyzed (Figure 5). AmNA[+21/+40] administration significantly ($p < 0.01$) reduced the tumor size, and these effects were enhanced by cRGD-conjugated AmNA[+21/+40] (Figures 5A–5D). Nevertheless, body weight did not considerably change in any group, and the AST/ALT levels on day 9 were almost the same in the cRGD-conjugated AmNA[+21/+40] and the saline and NC groups (Figures 5B and 5C), suggesting low toxicity. The quantification of REST_SSO revealed increased REST_SSO levels in tumors after cRGD-conjugated AmNA[+21/+40] administration, as determined by modified enzyme-linked oligosorbent assay (ELOSA)[37] (Figure 5E). ELOSA was performed to quantify the oligonucleotide-specific sequence; the standard curve in the range of 0.01–0.1 nM was used in this study (Figure S7). Together, we observed that cRGD-conjugated AmNA[+21/+40] improved the efficacy in terms of its antitumor effects, possibly owing to the effective uptake of this SSO into the tumor. Intratumoral *REST* splicing analysis was not performed because the tumor was dramatically shrunk and a sufficient amount of RNA could not be obtained. Increased cRGD-conjugated AmNA[+21/+40] in tumors may be due to the higher expression of the cRGD receptor integrin β1 in tumors and lower expression in the livers and kidneys.

Microarray analysis of mRNAs and miRNAs in 22Rv1 cells transfected with AmNA[+21/+40] and AmNA[+23/+44]

Exon skipping activity, cell viability, and *in vivo* activity by REST_SSO were analyzed in 22Rv1 cells. AmNA[+21/+40] and AmNA[+23/+44] were transfected into 22Rv1 cells at 10 nM using lipofection. Total RNA was extracted after 48 h, followed by microarray analysis. Based on the results of a data analysis with changes of over 2-fold or less than half in the expression compared with the NC, the expression of 55 and 8 genes changed in the AmNA[+21/+40] and AmNA[+23/+44] groups, respectively (Figure S8; Table S3). All significantly altered genes were analyzed and compared with RE1 genes by referencing the ChIP-Atlas database (https://chip-atlas.org/).[38] Of 43 genes with considerably decreased expression due to AmNA[+21/+40] and AmNA[+23/+44], 41 were identified as REST-controlled genes.[39,40] The functions of two uncharacterized downregulated genes (LOC107985773 and KIAA0408) have not been widely reported. The reason for one RE1 gene (*INSM2*) that was downregulated by AmNA[+21/+40] but not by AmNA [+23/+44] may be attributed to the lower exon skipping activity of AmNA[+23/+44] than of AmNA[+21/40]. Ten genes upregulated by AmNA[+21/+40] were not RE1 genes, whereas two downregulated genes were RE1 genes. Five genes upregulated by AmNA [+23/+44] were not RE1 genes, whereas three downregulated genes were RE1 genes. The *REST* level was slightly increased due to splicing changes induced by REST_SSO, and the two other genes (*DNAI7* and *CDKN1A*) are under investigation. *SRRM4* expression was substantially reduced by AmNA[+21/+40] and slightly reduced by AmNA[+23/+44], which is consistent with our previously published data.[20] The data obtained by microarray analysis were consistent; however, future comparative quantification analysis reveals the effects of REST_SSO. Next, the PCa cell line 22Rv1 was transfected with SRRM4_ASO, AmNA[+21/+40], or AmNA[+23/+44], and total RNA was analyzed for miRNA expression using microarray analysis (Figure S8; Table S4). Eight miRNAs, whose expression changed by more than 2-fold or less than half as compared with the NT control, were obtained because the NCs for REST_SSO and SRRM4_ASO were different. Five miRNAs were downregulated by SRRM4_ASO (AmNA7168) and REST_SSOs (AmNA[+21/+40] and AmNA[+23/+44]), whereas 14 miRNAs, including miR-4516, were considerably upregulated. We have reported that *SRRM4* expression in SCLC is induced by the reduced expression of intracellular miR-4516 through exosome secretion. miR-4516 is a useful biomarker for SCLC patients with higher serum miR-4516 concentrations.[17] Other miRNAs are under investigation. Notably, the most upregulated miR-5703 has been reported to increase after treating PCa PC-3 cells with luteolin, gefitinib, or both.[41] Furthermore, overexpressed miR-5703 has been shown to inhibit pressure-induced growth and metastasis in liver cancer.[42]

or NCI-H146 was transfected with 100 nM AmNA[+21/+40] or AmNA[+23/+44] using Lipofectamine, followed by RT-PCR. Exon skipping activity of each oligonucleotide has been shown as compared with that obtained for the NT control, which was set as 1 (n = 3, mean ± SEM). mock: lipofection without SSO; NC: NC oligonucleotide. Statistical significance was analyzed as compared with the values for the NT using 1-way ANOVA followed by Dunnett's t test. *$p < 0.05$; **$p < 0.01$; ***$p < 0.001$.

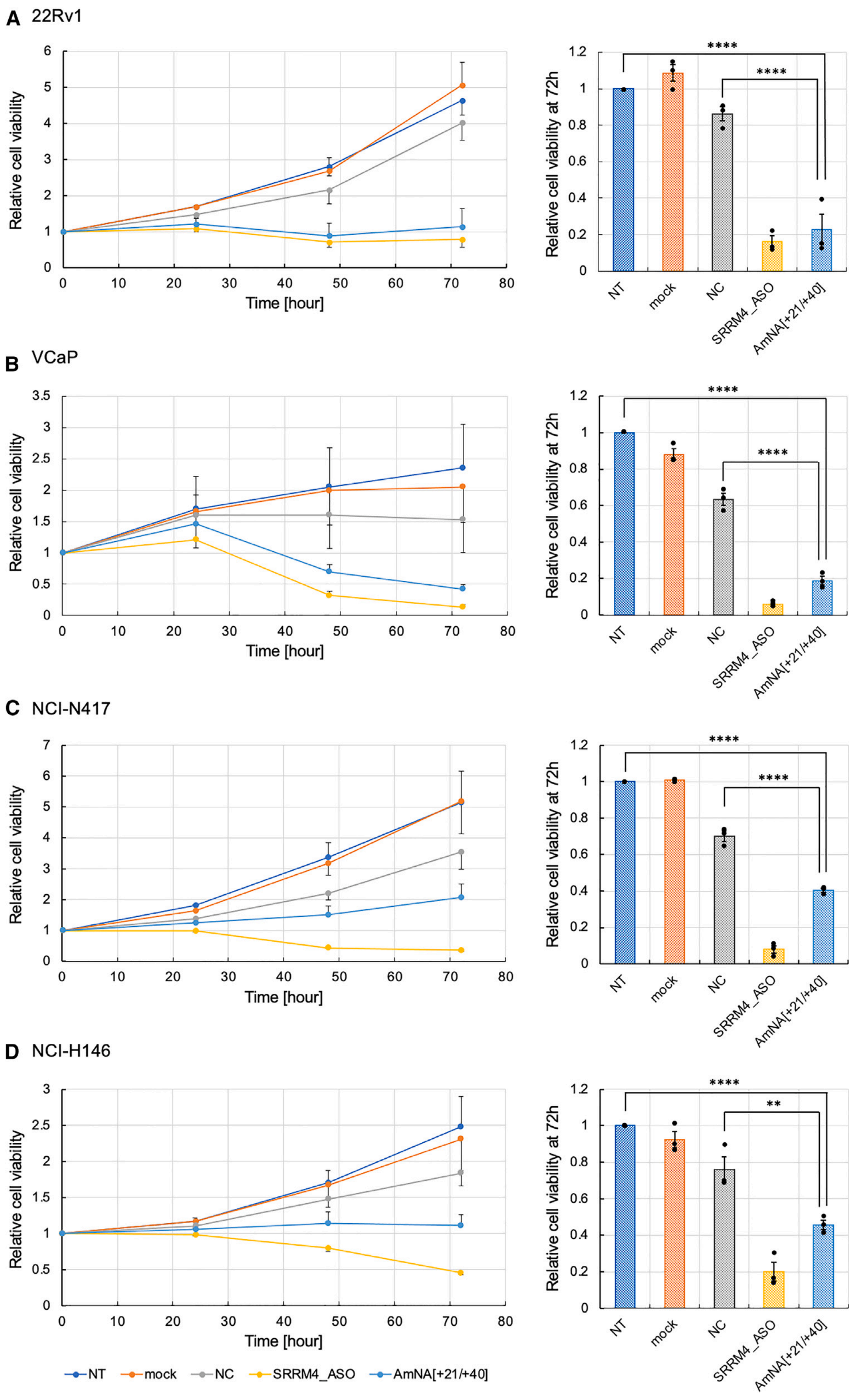

(legend on next page)

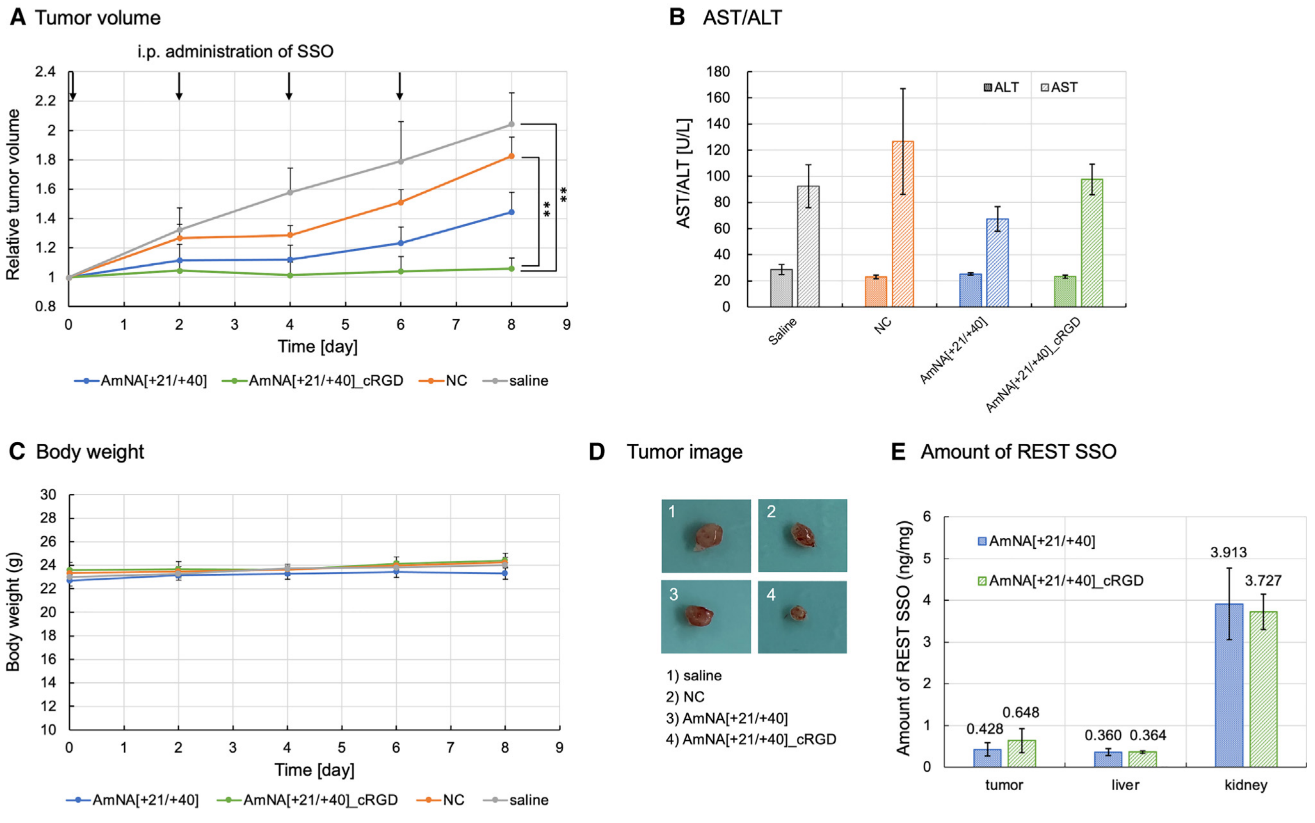

Figure 5. Antitumor effects after AmNA[+21/+40] administration in xenograft mice bearing tumors originating from the PCa cell line 22Rv1

(A) 22Rv1 cells (5.0×10^5) were subcutaneously implanted in 8-week-old BALB/c Slc-nu/nu mice (n = 5). After 7 days, each oligonucleotide or saline was intraperitoneally administered at 10 mg/kg every 2 days. Tumor volume was analyzed every 2 days until day 8. Statistical significance was analyzed as compared with the values for the NT using 1-way ANOVA followed by Dunnett's t test. $^{**}p < 0.01$. (B) AST/ALT test using blood samples. The blood obtained from each mouse on day 9 was used for the ALT/AST assay. (C) Body weight was assayed every 2 days. (D) Images of tumors obtained in different groups. (E) REST_SSO levels in the tumors, livers, and kidneys were analyzed using ELOSA.

DISCUSSION

We successfully developed a REST_SSO (AmNA[+21/+40]) targeting microexon N, which switches the splicing of the truncated form of *REST* without DNA-binding ability to the functional full-length *REST*. REST_SSO with AmNA[18,32] modifications appeared to achieve high activity because of screening and optimization processes. AmNA has also been used in an ASO targeting α-synuclein to treat Parkinson disease.[43] Abnormal expression of SRRM4 produces *sREST* mRNA in SCLC and PCa cells, which may underlie the pathogenesis of NE cancers.[7,44,45] Recently, the gapmer SRRM4_ASO was reported to reduce *SRRM4* expression in SCLC and PCa cells, wherein *REST* splicing was switched to *REST* mRNA from *sREST* mRNA.[20] Although we attempted to analyze the REST protein using several commercially available antibodies, an antibody targeting N-terminal REST failed to detect C-terminally truncated sREST and REST proteins due to the possibility of low antibody specificity. In the future, we will analyze REST protein expression using antibodies currently under production. In the present study, REST_ SSO administration (AmNA[+21/+40]) successfully reduced the proliferation of PCa cells (Figure 4) and exerted antitumor effects in xenograft mice (Figures 5, S5, and S6). REST_SSO may function equally well for both SCLC and PCa showing abnormal *REST* splicing. The cRGD-conjugated NC was employed for several *in vivo* antitumor analyses using SRRM4_ASO in 22Rv1 cells and had no antitumor effects and SRRM4 expression in xenograft mice (data not shown), suggesting that cRGD alone does not affects 22Rv1 tumors.

SSOs generally bind to pre-mRNAs and regulate splicing by competitively inhibiting their binding to splicing-related factors, including

Figure 4. Cell viability analysis of human SCLC and PCa cell lines after transfection with AmNA[+21/+40]

Data for (A) 22Rv1, (B) VCaP, (C) NCI-N417, and (D) NCI-H146 cell lines have been shown. Every cell line (5.0×10^3) was transfected with 100 nM oligonucleotide (for VCaP, N417, and H146) and 50 nM oligonucleotide (for 22Rv1) using Lipofectamine 3000 and cultured for 72 h. Cell viability was quantified every 24 h. Mock: transfection reagent only. SRRM4_ASO has been shown to reduce cell viability in a previous study.[20] Statistical significance was analyzed as compared with the values for the NT using 1-way ANOVA followed by Dunnett's t test. $^{**}p < 0.01$; $^{****}p < 0.0001$.

SRSFs. SSOs do not require mRNA cleavage by endogenous RNase H; thus, the use of natural nucleic acids in SSOs to form the gap region is unnecessary, as in gapmer ASO. Hence, SSOs generally adopt a mixmer structure, wherein modified and natural nucleic acids are arranged alternately.[29] Since 2022, five SSOs have been approved as pharmaceutical medicines and have been focused on as alternative approaches for treating intractable diseases, including nusinersen (Spinraza) for treating spinal muscular atrophy[46] and viltolarsen (Viltepso) for treating Duchenne muscular dystrophy.[47] However, no SSOs have been approved as therapeutic anticancer medicines. This preclinical study paves the way for future studies intending to demonstrate the feasibility of using REST_SSO as an antitumor medicine for SCLC and PCa with NE phenotypes.

Microarray analysis of genes in 22Rv1 cells after REST_SSO administration revealed that many genes were substantially repressed. Most of these genes are reported to be REST-bound genes in the ChIP-Atlas and contain the RE1 element. This correlation supports the possibility that the REST protein was re-expressed upon treatment with REST_SSO. We attempted to detect the presumed sREST protein using several commercially available anti-REST antibodies that target the N-terminal of the REST protein; however, we did not observe any discernible bands, possibly due to the absence of protein expression. One possibility is that mRNA containing microexon N may be processed via NMD,[11,12] resulting in the loss of REST function. After Coulson et al.[5] reported that the REST isoform (sREST) was abnormally expressed in SCLC cells, we conducted experiments using cycloheximide; however, most SCLC cells showed reduced viability within 24 h. Similar to neurons, in differentiated SCLC cells, due to decreased REST expression, SCLC cells reduced cell viability by recovering REST expression. We previously reported that the exogenous REST expression in SCLC cells reduced viability.[20] To further understand REST_SSO function, we tried to quantitate REST or sREST mRNAs using RT-qPCR; however, this failed because the sequence around microexon N is quite similar. Thus, we are developing new quantification methods. Exogenous expression of the deduced sREST protein has previously resulted in a loss-of-function REST without a DNA-binding domain and resulted in function as a dominant negative.[10] Loss of REST function is associated with a more aggressive phenotype in breast cancer,[9] as well as a worse prognosis in SCLC and NEPCa.[5,7] Using mRNA microarray analysis, we observed the downregulation of *SRRM4* mRNA, a splicing activator of REST, to 0.77 upon AmNA[+21/+40] transfection in 22Rv1 cells, compared with NC transfection. Furthermore, *SRRM3* levels,[48] another SRRM4 ortholog, were reduced by the RE1-mediated mechanism upon REST_SSO transfection (Table S3). In addition, miRNA microarray analysis revealed that 8 miRNAs, including miR-4516, were considerably upregulated by either REST_SSO (AmNA [+21/+40] or AmNA[+23/+44]) or SRRM4_ASO (AmNA7168) (Table S3). We previously reported that miR-4516 is an underlying reason for the abnormal expression of SRRM4 and is a useful plasma biomarker for patients with SCLC.[17] miR-4516 expression predicts poor prognosis and is substantially increased in the serum of healthy individuals living in a city moderately polluted by PM (particulate matter) 2.5.[49] The role of miR-4516 has also been reported in cancer progression.[50–52] Intracellular miR-4516 in 22Rv1 cells increased after REST_SSO treatment, possibly because the number of cells expressing SRRM4 was reduced or cells, such as cancer stem cells, expressed miR-4516. This should be addressed further to determine the therapeutic effectiveness of REST_SSO and to evaluate miR-4516 as a therapeutic biomarker. Based on the microarray analysis results, we need to further analyze the gene functions and miRNA changes facilitating the therapeutic effects of REST_SSO and SRRM4_ASO. In addition, we need to further analyze tumor gene expression via microarray analysis to reveal the function of REST_SSO *in vivo*. SCLC exhibits high tumor heterogeneity.[33,34] The heterogeneity of the NE transcriptional state is highly affected in metastatic SCLC and patient-derived models.[53] Although tumor heterogeneity may make it challenging to treat SCLC or NEPCa, our SRRM4_ASO and REST_SSO—possibly combined with other treatments—may be effective therapeutic options. One reason could be that abnormal *REST* splicing may be caused by *SRRM4* or *SRRM3*.

Based on our study, REST_SSO can be formulated as an antitumor medicine and is expected to be applied to other diseases involving REST, such as heart failure.[54,55] The splicing regulation of *REST* can be targeted for treating many diseases. REST is involved in the expression of the heart failure markers ANP and BNP.[56] GNAO1, which was downregulated by REST, has been reported recently to be a therapeutic target of heart failure.[57] Loss of REST function is observed in approximately 20% of breast cancer cases and is associated with an aggressive phenotype and poor prognosis.[9] REST target gene upregulation due to the loss of REST leads to aberrant signaling and tumor pathogenesis in NE cancers, aggressive breast cancer types, SCLC, and PCa.[58–60] Altogether, REST_SSO may be a good therapeutic option, whereas SRRM4_ASO is an alternative for exhibiting quicker pharmacological effects for various cancers. Because SRRM4_ASO and REST_SSO have different mechanisms of action, their use and safety should be verified for individual patients. Our findings suggest that controlling the alternative splicing of *REST* by either REST_SSO or the previously developed SRRM4_ASO may prove to be an effective antitumor medicine for treating SCLC and PCa. To realize the therapeutic applications of REST_SSO, we need to further analyze its long-term effects using the Kaplan-Meier method. Oligonucleotide medicine exhibits lower retention in the blood, along with limited tumor targeting. Our cRGD-AmNA[+21/+40] improved cellular uptake and antitumor effects; however, a drug delivery system, such as one that reduces retention in the liver and kidneys, is needed for better safety. Currently, we are optimizing appropriate ligands to develop better pharmaceutical medicine.

MATERIAL AND METHODS

Cell culture

All cell lines were obtained from the American Type Culture Collection (Manassas, VA). The following cell lines were used in this study: SCLC cell lines STC-1 (CRL-3254), NCI-H146 (HTB-173), and NCI-N417 (CRL-5809), and PCa cell lines 22Rv1 (CRL-2505) and VCaP (CRL-2876). All PCa and STC-1 cells were cultured as adherent cells,

whereas other SCLC cell lines were cultured as floating aggregates in RPMI-1640 medium (catalog no. 187–02705; FUJIFILM Wako Pure Chemical, Osaka, Japan) containing 10% fetal bovine serum (catalog no. 10270106; Thermo Fisher Scientific, Waltham, MA). The cells were cultured at 37°C in a humidified incubator with 5% CO_2.

Oligonucleotide synthesis

Functional SSOs were selected after *in silico* screening of the oligonucleotides around microexon N in the human *REST* sequence. SSO is an 18-mer phosphorothioate oligonucleotide containing AmNA nucleosides in alternating positions with deoxynucleosides, starting with a 3′-deoxynucleoside. These modifications contribute to improved target sequence affinity, nuclease resistance, and low toxicity. Oligonucleotides were synthesized by Aji Bio-Pharma (Osaka, Japan), were purified using high-performance liquid chromatography, and confirmed using mass analysis. SSO sequences used in this study are listed in Table S1.

Measurement of the T_m of oligonucleotides

The UV melting experiments were conducted using the UV-1900i spectrophotometer equipped with T_m analysis accessory TMSPC-8 (Shimadzu, Kyoto, Japan). SSO and equimolecular amounts of cRNA oligonucleotide (RNA1948: UGGUAAUAUUUACUAGAGU GUGAUCUAGAU) were dissolved in 10 mM sodium phosphate buffer (pH 7.2) containing 10 mM NaCl to obtain a final strand concentration of 1.0 μM. Samples were boiled and cooled slowly to room temperature. The UV absorption at 260 nm was recorded from 5°C to 90°C at a scan rate of 0.5°C/min. The first derivative was calculated using a smoothed UV melting profile. The peak temperatures in the derivative curve were designated as the T_m.

In vitro SSO transfection

STC-1 cells (1.0×10^6/well) were transfected using the Neon Transfection System (Thermo Fisher Scientific). Briefly, various amounts of SRRM4_ASO (nmol/1.0×10^6 cells) and REST_SSOs shown in each figure were used in 10 or 100 μL in the tip for the electroporation system. The electroporation conditions were as follows: pulse voltage of 1,200 V, pulse width of 20 ms, and pulse number of 2 pulses. After transfection, the cells were cultured in 2 mL medium in a 6-well plate (catalog no. 3810-006; IWAKI, Shizuoka, Japan) for 24 h. After optimization, REST_SSOs were transfected into the cells (confluence of 70%–80%) using Lipofectamine 3000 (catalog no. L3000015; Thermo Fisher Scientific). Briefly, the cells (1.0×10^5/well) cultured on 24-well plates (catalog no. 3820-024; IWAKI) were incubated with 1.5 μL Lipofectamine 3000 in 500 μL medium with various amounts of REST_SSOs. The cells were cultured at 37°C in a humidified incubator with 5% CO_2. For the cell viability assay, cells (5,000/well) cultured on a 96-well microplate (catalog no. 3917; Corning, Corning, NY) were incubated with various amounts of REST_SSOs with 0.09 μL Lipofectamine 3000 in 50 μL medium. The cells were cultured at 37°C in a humidified incubator with 5% CO_2 for 48 h.

RT-PCR

Total RNA was prepared using the RNeasy Plus Micro Kit (Qiagen, Germantown, MD). Total RNA was spectrophotometrically quantified at 260 nm using a spectrophotometer (DS-11; DeNovix, Wilmington, DE) and was verified to be of high quality. The quality of a few RNA samples prepared using the same methodology was assessed using a bioanalyzer (Agilent, Santa Clara, CA), wherein the RNA integrity number (RIN) was found to be mostly >7.0. Total RNA (200 or 500 ng) was transcribed at 42°C for 60 min using the SuperScript IV VILO kit (catalog no. 11756500; Thermo Fisher Scientific). cDNA was amplified using Hot Start Taq DNA Polymerase (M0495L; NEB, Ipswich, MA). Amplification was performed using a Dice Touch PCR Thermal Cycler (Takara Bio, Shiga, Japan). PCR was conducted with initial activation at 95°C for 30 s, followed by 20 (β-actin), 27 (for PCa cells), or 33 cycles (for SCLC cells) (REST and sREST) of amplification (95°C for 15 s, 58°C for 15 s, and 68°C for 30 s), and 68°C for 2 min. Forward and reverse primers (5 pmol each) were used in 25-μL reaction mixtures. The primer sequences were as follows: REST forward: 5′-GAACGCCCATATA AATGTGAA-3′; REST reverse: 5′-TTTGAAGTTGCTTCTATCTG CTGT-3′; β-actin forward: 5′-GGCCGTCTTCCCCTCCATCG-3′; and β-actin reverse: 5′-CCAGTTGGTGACGATGCCGTGC-3′. PCR products were analyzed by electrophoresis using 5% Mini-PROTEAN gels (catalog no. 4565016B02; Bio-Rad, Hercules, CA) at 100 V for 40 min, followed by ethidium bromide staining. The gel images were obtained at an exposure time of 0.5 s using the iBright Imaging system (Thermo Fisher Scientific). The intensity of each band was quantified using ImageJ software (http://imagej.nih.gov/ij/). Each band was excised, and the sequences were analyzed to confirmation by Sanger sequencing. The percentage of exclusion was calculated as the band intensity of sREST against the total band intensities of REST and sREST. The following formula was used: Exon skipping activity = [REST band intensity]/[REST band intensity] + [sREST band intensity].

Cell viability assay

Cell viability was measured using the Cell Counting Kit-8 (catalog no. 343–07623; Dojindo, Kumamoto, Japan) or the CellTiter-Glo 3D cell viability assay (catalog no. G968A, Promega, Madison, WI), according to the manufacturer's protocol. After culturing the treated and untreated cells in a 96-well plate for an appropriate culture time, 50 μL CellTiter-Glo reagent was added, and the plates were shaken for 30 s using BioShake XP (QInstruments, Jena, Germany). The assay was performed more than three times to confirm reproducibility. The luminescence signal was analyzed using the Infinite M1000 (Tecan, Männedorf, Switzerland) or the Nivo Multimode Plate Reader (PerkinElmer, Waltham, MA).

Animals

All animal procedures were performed according to the protocol approved by the Animal Experimentation Committee of Osaka University. Athymic nude mice (7-week-old male BALB/c Slc-nu/nu) were obtained from Shimizu Laboratory Supplies (Kyoto, Japan) and allowed to acclimatize for 1 week before the experiments. All mice used in this study were housed in AAALAC-accredited facilities, and the overall protocol was approved by the Institutional Care and Use Committee of Osaka University (no. R05-1-1). The environment

was maintained at 21°C ± 1°C with 50% ± 20% relative humidity, and the rooms were ventilated using a minimum of 15 HEPA-filtered air changes per hour. The animals were maintained under a 12:12 h light/dark cycle and provided *ad libitum* water and feed.

In vivo tumor formation analysis

N417 cells were treated with Accutase for 5 min (catalog no. AT104; Funakoshi, Tokyo, Japan), and 22Rv1 cells were harvested with trypsin (catalog no. 35554-64; Nacalai Tesque, Kyoto, Japan). Cells were collected by centrifugation at 1,000 × *g* for 3 min. An aliquot of cells disaggregated by gentle trituration was used to assess cell viability and number. Cells were suspended in 50 μL serum-free RPMI-1640 medium, and 50 μL Matrigel (catalog no. 354234; Corning) was mixed. N417 cells (5.0×10^5) or 22Rv1 cells (1.0×10^6) were implanted subcutaneously into the mid-dorsal region of every 7-week-old male nude mouse (BALB/c Slc-nu/nu) under isoflurane anesthesia (catalog no. 099–06571; FUJIFILM Wako Pure Chemical). Tumors were allowed to grow for 1–2 weeks and reach sizes of 30–50 mm^3. Subsequently, SSOs (10.0 mg/kg) or saline (control) were intraperitoneally administered 4 times every 2 or 3 days. One to three days after the final dose of SSOs, the tumor and blood were collected. The tumor was immediately immersed in 700 μL QIAzol Lysis Reagent (catalog no. 79306; Qiagen) and 140 μL chloroform (catalog no. 08402-84; Nacalai Tesque), and total RNA was then extracted as mentioned above. For *in vivo* tumor analysis, long and short diameters were measured, and tumor volume was calculated as follows: 0.5 × (tumor long diameter) × (tumor minor diameter^2).

Hepatotoxicity assessment

Blood samples were collected via cardiac puncture from mice after SSO administration and allowed to stand overnight at 4°C. The samples were centrifuged at 3,000 relative centrifugal force for 5–10 min to obtain serum; 10 μL serum was assayed. Serum AST and ALT levels were measured using DRI-CHEM 4000V (FUJIFILM Wako Pure Chemical) using DRI-CHEM Slides GPT/ALT-PIII (catalog no. 14A2X10004-000010) and GOT/AST-PIII (catalog no. 14A2X10004-000009).

ELOSA

Tumors and organs (livers and kidneys) after SSO administration were collected and stored in a freezer at −80°C. Tissue lysate was obtained by homogenizing 20–30 mg of tissues in radioimmunoprecipitation assay buffer (2.5 μL/mg) (catalog no. 08714-04; Nacalai Tesque). The tissue suspension was diluted with Dulbecco's PBS (DPBS; catalog no. 166–23555; FUJIFILM Wako Pure Chemical). A streptavidin-coated 96-well plate (catalog no. 15503; Thermo Fisher Scientific) was washed with 120 μL DPBS containing Tween 20 (PBS-T; catalog no. 170–0531; Bio-Rad) twice. Template solution (100 μL) containing AmNA[+21/+40]_template was diluted with PBS-T (×1,000) and added into each well, followed by incubation at 37°C for 2 h. A standard curve using known concentrations of AmNA[+21/+40] added to normal tissue lysate was prepared using a four-parameter logistic analysis method (Figure S7). After washing twice with 120 μL PBS-T, 100 μL freshly prepared T4 DNA ligase (catalog no. B0202S; NEB) was added and incubated at 15°C overnight. Wells were then washed two times with SuperBlock blocking buffer (catalog no. 37580; Thermo Fisher Scientific) prepared in DPBS (b-PBS), followed by treatment with 40 μL S1 nuclease solution (catalog no. 2410A; Takara Bio) for 30 min. Then, 100 μL anti-digoxigenin-AP Fab fragment solution (catalog no. 11093274910; Roche, Buchs, Switzerland) was added and incubated at 37°C for 1.5 h in a humidified incubator with 5% CO_2. After washing two times with b-PBS, 100 μL AttophosAP (catalog no. S1001; Promega) was added, and fluorescence emission intensity was measured after 30 min using the Nivo Multimode Plate Reader (PerkinElmer). AmNA[+21/+40] in the tissue was quantified based on the luminescence intensity using a standard curve.

In silico data analysis

The higher-order structure prediction for each SSO was performed using the following websites:

- RNAfold Webserver (http://rna.tbi.univie.ac.at/cgi-bin/RNAWebSuite/RNAfold.cgi)
- UNAfold Webserver (http://www.unafold.org/mfold/applications/rna-folding-form-v2.php)

Briefly, the formation of higher-order structures by intramolecular interactions was predicted based on each sequence, whereas the intermolecular interaction was analyzed using SSO linked to a 5-base DNA linkage (SSO-NNNNN-SSO). Off-target genes interacting with each SSO were analyzed with the GGGenome Database using SSO sequences while allowing 0–2 mismatches and gaps.

Microarray analysis

The 22Rv1 (1.0×10^6) cells were transfected with 10 nM AmNA[+21/+40], AmNA[+23/+44], NC, or AmNA7168 using Lipofectamine 3000 (Thermo Fisher Scientific). After 48 h of transfection, RNA was extracted, and total RNA was quantified by measuring the absorbance at 260 nm. The quality of RNA was >1.9 at 260/280 nm, and the RIN was >8.1, as estimated using 4200 TapeStation (Agilent). Microarray analysis was performed using Agilent kits for mRNA (SurePrint G3 8 × 60 K version 3.0 Human GE Microarray) and miRNA (SurePrint G3 Human miRNA). Slides were scanned immediately after washing on the Agilent DNA Microarray Scanner (G2505B). The scanned images were analyzed with Feature Extraction Software version 12.1.1.1 (Agilent) using default parameters (protocol miRNA_1200_Jun14 and Grid: 070156_D_F_20141006, protocol GE1_1200_Jun14 and Grid: 072363_D_F_20221108) to obtain background-subtracted and spatially detrended processed signal intensities. Raw data were normalized to the 75th percentile (mRNA) or the 90th percentile signal intensity (miRNA), as recommended by the vendor. Microarray analysis was conducted by the Chemical Evaluation and Research Institute (Tokyo, Japan).

All data were deposited to GEO and are available under the accession number GSE245707. The ChIP-Atlas database was used to identify the reference genes regulated by REST.

Statistical analysis

Each time point was assayed at least in triplicate, and all experiments were performed multiple times to confirm reproducibility (n = 3). Data are represented as mean ± SEM obtained from three to five independent experiments. Statistical differences were analyzed using one-way ANOVA, followed by Dunnett's or Tukey's t test. The following statistically significant differences were considered: *$p < 0.05$; **$p < 0.01$; ***$p < 0.001$; ****$p < 0.0001$.

DATA AND CODE AVAILABILITY

The data underlying this article will be shared upon reasonable request to the corresponding author.

SUPPLEMENTAL INFORMATION

Supplemental information can be found online at https://doi.org/10.1016/j.omtn.2024.102250.

ACKNOWLEDGMENTS

We are grateful to everyone at Bioorganic Chemistry Laboratory who discussed the data and advised us regarding the experiments. This work was supported by AMED (grant no. JP19am0401003), Japan; the Research Support Project for Life Science and Drug Discovery (Basis for Supporting Innovative Drug Discovery and Life Science Research) from AMED (grant nos. JP22ama121052 and JP22ama121054), Japan; JSPS KAKENHI (grant no. 22K07230), Japan; and the Osaka University Innovation Bridge (grant no. J210901039), Japan.

AUTHOR CONTRIBUTIONS

Conceptualization, K.M., S.O., and M.S. Formal analysis, K.M., S.O., and M.S. Methodology, K.M., S.O., and M.S. Validation, K.M., S.O., and M.S. Writing – original draft, K.M. and M.S. Writing – review & editing, K.M., S.O., and M.S.

DECLARATION OF INTERESTS

The authors declare no competing interests.

REFERENCES

1. Kawasaki, K., Rekhtman, N., Quintanal-Villalonga, Á., and Rudin, C.M. (2023). Neuroendocrine neoplasms of the lung and gastrointestinal system: convergent biology and a path to better therapies. Nat. Rev. Clin. Oncol. *20*, 16–32.
2. Cheng, S., Yang, S., Shi, Y., Shi, R., Yeh, Y., and Yu, X. (2021). Neuroendocrine prostate cancer has distinctive, non-prostatic HOX code that is represented by the loss of HOXB13 expression. Sci. Rep. *11*, 2778.
3. Mozzi, A., Guerini, F.R., Forni, D., Costa, A.S., Nemni, R., Baglio, F., Cabinio, M., Riva, S., Pontremoli, C., Clerici, M., et al. (2017). REST, a master regulator of neurogenesis, evolved under strong positive selection in humans and in non human primates. Sci. Rep. *7*, 9530.
4. Wang, G., Yang, X., Qi, M., Li, M., Dong, M., Xu, R., and Zhang, C. (2023). Systematic analysis identifies REST as an oncogenic and immunological biomarker in glioma. Sci. Rep. *13*, 3023.
5. Coulson, J.M. (2005). Transcriptional regulation: cancer, neurons and the REST. Curr. Biol. *15*, R665–R668.
6. Shimojo, M., Shudo, Y., Ikeda, M., Kobashi, T., and Ito, S. (2013). The small cell lung cancer-specific isoform of RE1-silencing transcription factor (REST) is regulated by neural-specific Ser/Arg repeat-related protein of 100 kDa (nSR100). Mol. Cancer Res. *11*, 1258–1268.
7. Li, Y., Zhang, Q., Lovnicki, J., Chen, R., Fazli, L., Wang, Y., Gleave, M., Huang, J., and Dong, X. (2019). SRRM4 gene expression correlates with neuroendocrine prostate cancer. Prostate *79*, 96–104.
8. Raj, B., O'Hanlon, D., Vessey, J.P., Pan, Q., Ray, D., Buckley, N.J., Miller, F.D., and Blencowe, B.J. (2011). Cross-regulation between an alternative splicing activator and a transcription repressor controls neurogenesis. Mol. Cell *43*, 843–850.
9. Cloud, A.S., Vargheese, A.M., Gunewardena, S., Shimak, R.M., Ganeshkumar, S., Kumaraswamy, E., Jensen, R.A., and Chennathukuzhi, V.M. (2022). Loss of REST in breast cancer promotes tumor progression through estrogen sensitization, MMP24 and CEMIP overexpression. BMC Cancer *22*, 180.
10. Shimojo, M., Lee, J.H., and Hersh, L.B. (2001). Role of zinc finger domains of the transcription factor neuron-restrictive silencer factor/repressor element-1 silencing transcription factor in DNA binding and nuclear localization. J. Biol. Chem. *276*, 13121–13126.
11. Kurosaki, T., Popp, M.W., and Maquat, L.E. (2019). Quality and quantity control of gene expression by nonsense-mediated mRNA decay. Nat. Rev. Mol. Cell Biol. *20*, 406–420.
12. Tan, K., Stupack, D.G., and Wilkinson, M.F. (2022). Nonsense-mediated RNA decay: an emerging modulator of malignancy. Nat. Rev. Cancer *22*, 437–451.
13. Bray, J.K., Elgamal, O.A., Jiang, J., Wright, L.S., Sutaria, D.S., Badawi, M., Borcyk, M.G., Liu, X., Fredenburg, K.M., Campbell-Thompson, M.L., and Schmittgen, T.D. (2020). Loss of RE-1 silencing transcription factor accelerates exocrine damage from pancreatic injury. Cell Death Dis. *11*, 138.
14. Zhang, X., Coleman, I.M., Brown, L.G., True, L.D., Kollath, L., Lucas, J.M., Lam, H.M., Dumpit, R., Corey, E., Chéry, L., et al. (2015). SRRM4 expression and the loss of REST activity may promote the emergence of the neuroendocrine phenotype in castration-resistant prostate cancer. Clin. Cancer Res. *21*, 4698–4708.
15. Chen, G.L., and Miller, G.M. (2018). Alternative REST splicing underappreciated. eNeuro *5*, 34–118.
16. Nakano, Y., Kelly, M.C., Rehman, A.U., Boger, E.T., Morell, R.J., Kelley, M.W., Friedman, T.B., and Bánfi, B. (2018). Defects in the alternative splicing-dependent regulation of REST cause deafness. Cell *174*, 536–548.e21.
17. Shimojo, M., Kasahara, Y., Inoue, M., Tsunoda, S.I., Shudo, Y., Kurata, T., and Obika, S. (2019). A gapmer antisense oligonucleotide targeting SRRM4 is a novel therapeutic medicine for lung cancer. Sci. Rep. *9*, 7618.
18. Yamamoto, T., Yahara, A., Waki, R., Yasuhara, H., Wada, F., Harada-Shiba, M., and Obika, S. (2015). Amido-bridged nucleic acids with small hydrophobic residues enhance hepatic tropism of antisense oligonucleotides in vivo. Org. Biomol. Chem. *13*, 3757–3765.
19. Shen, X., and Corey, D.R. (2018). Chemistry, mechanism and clinical status of antisense oligonucleotides and duplex RNAs. Nucleic Acids Res. *46*, 1584–1600.
20. Yoshida, M., Oda, C., Mishima, K., Tsuji, I., Obika, S., and Shimojo, M. (2023). An antisense amido-bridged nucleic acid gapmer oligonucleotide targeting SRRM4 alters REST splicing and exhibits anti-tumor effects in small cell lung cancer and prostate cancer cells. Cancer Cell Int. *23*, 8.
21. Dugger, S.A., Platt, A., and Goldstein, D.B. (2018). Drug development in the era of precision medicine. Nat. Rev. Drug Discov. *17*, 183–196.
22. Amanat, M., Nemeth, C.L., Fine, A.S., Leung, D.G., and Fatemi, A. (2022). Antisense oligonucleotide therapy for the nervous system: from bench to bedside with emphasis on pediatric neurology. Pharmaceutics *14*, 2389.
23. Khurshid, S., Montes, M., Comiskey, D.F., Shane, B., Matsa, E., Jung, F., Brown, C., Bid, H.K., Wang, R., Houghton, P.J., et al. (2022). Splice-switching of the insulin receptor pre-mRNA alleviates tumorigenic hallmarks in rhabdomyosarcoma. npj Precis. Oncol. *6*, 1.
24. Lee, Y., and Rio, D.C. (2015). Mechanisms and regulation of alternative pre-mRNA splicing. Annu. Rev. Biochem. *84*, 291–323.
25. Roberts, T.C., Langer, R., and Wood, M.J.A. (2020). Advances in oligonucleotide drug delivery. Nat. Rev. Drug Discov. *19*, 673–694.
26. Stanley, R.F., and Abdel-Wahab, O. (2022). Dysregulation and therapeutic targeting of RNA splicing in cancer. Nat. Cancer *3*, 536–546.
27. Bradley, R.K., and Anczuków, O. (2023). RNA splicing dysregulation and the hallmarks of cancer. Nat. Rev. Cancer *23*, 135–155.

28. Zhang, Y., Qian, J., Gu, C., and Yang, Y. (2021). Alternative splicing and cancer: A systematic review. Signal Transduct. Target. Ther. *6*, 78.

29. Shimo, T., Nakatsuji, Y., Tachibana, K., and Obika, S. (2021). Design and in vitro evaluation of splice-switching oligonucleotides bearing locked nucleic acids, amido-bridged nucleic acids, and guanidine-bridged nucleic acids. Int. J. Mol. Sci. *22*, 3526.

30. Østergaard, M.E., De Hoyos, C.L., Wan, W.B., Shen, W., Low, A., Berdeja, A., Vasquez, G., Murray, S., Migawa, M.T., Liang, X.H., et al. (2020). Understanding the effect of controlling phosphorothioate chirality in the DNA gap on the potency and safety of gapmer antisense oligonucleotides. Nucleic Acids Res. *48*, 1691–1700.

31. Vester, B., and Wengel, J. (2004). LNA (locked nucleic acid): high-affinity targeting of complementary RNA and DNA. Biochemistry *43*, 13233–13241.

32. Shimo, T., Tachibana, K., Kawawaki, Y., Watahiki, Y., Ishigaki, T., Nakatsuji, Y., Hara, T., Kawakami, J., and Obika, S. (2019). Enhancement of exon skipping activity by reduction in the secondary structure content of LNA-based splice-switching oligonucleotides. Chem. Commun. (Camb) *55*, 6850–6853.

33. Stewart, C.A., Gay, C.M., Xi, Y., Sivajothi, S., Sivakamasundari, V., Fujimoto, J., Bolisetty, M., Hartsfield, P.M., Balasubramaniyan, V., Chalishazar, M.D., et al. (2020). Single-cell analyses reveal increased intratumoral heterogeneity after the onset of therapy resistance in small-cell lung cancer. Nat. Cancer *1*, 423–436.

34. Yang, D., Denny, S.K., Greenside, P.G., Chaikovsky, A.C., Brady, J.J., Ouadah, Y., Granja, J.M., Jahchan, N.S., Lim, J.S., Kwok, S., et al. (2018). Intertumoral heterogeneity in SCLC Is influenced by the cell type of origin. Cancer Discov. *8*, 1316–1331.

35. Quader, S., Liu, X., Chen, Y., Mi, P., Chida, T., Ishii, T., Miura, Y., Nishiyama, N., Cabral, H., and Kataoka, K. (2017). cRGD peptide-installed epirubicin-loaded polymeric micelles for effective targeted therapy against brain tumors. J. Control. Release *258*, 56–66.

36. Liu, X., Wang, W., Samarsky, D., Liu, L., Xu, Q., Zhang, W., Zhu, G., Wu, P., Zuo, X., Deng, H., et al. (2014). Tumor-targeted in vivo gene silencing via systemic delivery of cRGD-conjugated siRNA. Nucleic Acids Res. *42*, 11805–11817.

37. Wei, X., Dai, G., Marcucci, G., Liu, Z., Hoyt, D., Blum, W., and Chan, K.K. (2006). A specific picomolar hybridization-based ELISA assay for the determination of phosphorothioate oligonucleotides in plasma and cellular matrices. Pharm. Res. (N. Y.) *23*, 1251–1264.

38. Oki, S., Ohta, T., Shioi, G., Hatanaka, H., Ogasawara, O., Okuda, Y., Kawaji, H., Nakaki, R., Sese, J., and Meno, C. (2018). ChIP-Atlas: A data-mining suite powered by full integration of public ChIP-seq data. EMBO Rep. *19*, e46255.

39. McGann, J.C., Spinner, M.A., Garg, S.K., Mullendorff, K.A., Woltjer, R.L., and Mandel, G. (2021). The genome-wide binding profile for human RE1 Silencing transcription factor unveils a unique genetic circuitry in hippocampus. J. Neurosci. *41*, 6582–6595.

40. Satoh, J.-I., Kawana, N., and Yamamoto, Y. (2013). ChIP-Seq data mining: remarkable differences in NRSF/REST target genes between human ESC and ESC-derived neurons. Bioinform. Biol. Insights *7*, 357–368.

41. Sakurai, M.A., Ozaki, Y., Okuzaki, D., Naito, Y., Sasakura, T., Okamoto, A., Tabara, H., Inoue, T., Hagiyama, M., Ito, A., et al. (2014). Gefitinib and luteolin cause growth arrest of human prostate cancer PC-3 cells via inhibition of cyclin G-associated kinase and induction of miR-630. PLoS One *9*, e100124.

42. Shen, S., Zhou, W., Xuan, J., Xu, W., Xu, H., Yang, M., Zhu, L., Yang, Z., Yang, B., Shi, B., et al. (2022). Overexpression of pressure-responsive miRNA-5703 inhibits pressure-induced growth and metastasis of liver cancer. J. Cancer *13*, 325–342.

43. Uehara, T., Choong, C.J., Nakamori, M., Hayakawa, H., Nishiyama, K., Kasahara, Y., Baba, K., Nagata, T., Yokota, T., Tsuda, H., et al. (2019). Amido-bridged nucleic acid (AmNA) -modified antisense oligonucleotides targeting α-synuclein as a novel therapy for Parkinson's disease. Sci. Rep. *9*, 7567.

44. Lin, Z., Radaeva, M., Cherkasov, A., and Dong, X. (2022). Lin28 regulates cancer cell stemness for tumour progression. Cancers *14*, 4640.

45. Radaeva, M., Ho, C.H., Xie, N., Zhang, S., Lee, J., Liu, L., Lallous, N., Cherkasov, A., and Dong, X. (2022). Discovery of novel Lin28 inhibitors to suppress cancer cell stemness. Cancers *14*, 5687.

46. Chilcott, E.M., Muiruri, E.W., Hirst, T.C., and Yáñez-Muñoz, R.J. (2022). Systematic review and meta-analysis determining the benefits of in vivo genetic therapy in spinal muscular atrophy rodent models. Gene Ther. *29*, 498–512.

47. Duan, D., Goemans, N., Takeda, S., Mercuri, E., and Aartsma-Rus, A. (2021). Duchenne muscular dystrophy. Nat. Rev. Dis. Primers *7*, 13.

48. Labrecque, M.P., Brown, L.G., Coleman, I.M., Lakely, B., Brady, N.J., Lee, J.K., Nguyen, H.M., Li, D., Hanratty, B., Haffner, M.C., et al. (2021). RNA splicing factors SRRM3 and SRRM4 distinguish molecular phenotypes of castration-resistant neuroendocrine prostate cancer. Cancer Res. *81*, 4736–4750.

49. Li, X., Lv, Y., Hao, J., Sun, H., Gao, N., Zhang, C., Lu, R., Wang, S., Yin, L., Pu, Y., and Chen, R. (2016). Role of microRNA-4516 involved autophagy associated with exposure to fine particulate matter. Oncotarget *7*, 45385–45397.

50. Hao, B., Shi, A., Li, X., Li, J., Liu, Z., and Yuan, H. (2020). miR-4516 inhibits the apoptosis of RB tumor cells by targeting the PTEN/AKT signaling pathway. Exp. Eye Res. *200*, 108224.

51. Jin, X.H., Lu, S., and Wang, A.F. (2020). Expression and clinical significance of miR-4516 and miR-21-5p in serum of patients with colorectal cancer. BMC Cancer *20*, 241.

52. Umair, Z., Baek, M.O., Song, J., An, S., Chon, S.J., and Yoon, M.S. (2022). MicroRNA-4516 in urinary exosomes as a biomarker of premature ovarian insufficiency. Cells *11*, 2797.

53. Lissa, D., Takahashi, N., Desai, P., Manukyan, I., Schultz, C.W., Rajapakse, V., Velez, M.J., Mulford, D., Roper, N., Nichols, S., et al. (2022). Heterogeneity of neuroendocrine transcriptional states in metastatic small cell lung cancers and patient-derived models. Nat. Commun. *13*, 2023.

54. Rogalska, M.E., Vivori, C., and Valcárcel, J. (2023). Regulation of pre-mRNA splicing: roles in physiology and disease, and therapeutic prospects. Nat. Rev. Genet. *24*, 251–269.

55. Marasco, L.E., and Kornblihtt, A.R. (2023). The physiology of alternative splicing. Nat. Rev. Mol. Cell Biol. *24*, 242–254.

56. Govindapillai, A., Hotchkiss, A., Baguma-Nibasheka, M., Rose, R.A., Miquerol, L., Smithies, O., Maeda, N., and Pasumarthi, K.B.S. (2018). Characterizing the role of atrial natriuretic peptide signaling in the development of embryonic ventricular conduction system. Sci. Rep. *8*, 6939.

57. Inazumi, H., Kuwahara, K., Nakagawa, Y., Kuwabara, Y., Numaga-Tomita, T., Kashihara, T., Nakada, T., Kurebayashi, N., Oya, M., Nonaka, M., et al. (2022). NRSF-GNAO1 pathway contributes to the regulation of cardiac Ca^{2+} homeostasis. Circ. Res. *130*, 234–248.

58. Arizmendi-Izazaga, A., Martínez-Baltazar, R., Liborio-Bautista, A., Olea-Flores, M., Ortiz-Ortiz, J., and Navarro-Tito, N. (2023). The NRSF/REST transcription factor in hallmarks of cancer: from molecular mechanisms to clinical relevance. Biochimie *206*, 116–134.

59. Belluti, S., Rigillo, G., and Imbriano, C. (2020). Transcription factors in cancer: when alternative splicing determines opposite cell fates. Cells *9*, 760.

60. Chen, G.L., and Miller, G.M. (2013). Extensive alternative splicing of the repressor element silencing transcription factor linked to cancer. PLoS One *8*, e62217.

OMTN, Volume *35*

Supplemental information

Splice-switching antisense oligonucleotide controlling tumor suppressor REST is a novel therapeutic medicine for neuroendocrine cancer

Keishiro Mishima, Satoshi Obika, and Masahito Shimojo

Supplemental Information

Table S1. Sequences of the oligonucleotides

Name	Oligonucleotide sequence (5'→ 3')	Base	Used in
AmNA[+31/+48]	A(Y)^t^5(Y)^t^A(Y)^g^A(Y)^t^5(Y)^a^5(Y)^a^5(Y)^t^5(Y)^t^A(Y)^g	18	Figure 1<br>Figures S1&2
AmNA[-20/-3]	G(Y)^g^T(Y)^c^5(Y)^a^A(Y)^t^G(Y)^g^A(Y)~a^T(Y)^g^5(Y)^a^T(Y)^a	18	Figure 1<br>Figures S1&2
AmNA[+19/+36]	5(Y)^t^5(Y)^t^A(Y)^g^T(Y)^a^A(Y)^a^T(Y)^a^T(Y)^t^A(Y)^c^5(Y)^a	18	Figure 1<br>Figures S1&2
AmNA[+5/+22]	A(Y)^c^5(Y)^a^A(Y)^a^T(Y)^g^G(Y)^t^A(Y)^t^5(Y)^c^A(Y)^t^A(Y)^c	18	Figure 1<br>Figures S1&2
AmNA[-24/-7]	5(Y)^a^A(Y)^t^G(Y)^g^A(Y)^a^T(Y)^g^5(Y)^a^T(Y)^a^G(Y)^t^A(Y)^g	18	Figure 1<br>Figure S2

AmNA[-18/-1]	5(Y)^t^G(Y)^g^T(Y)^c^5(Y)^a^A(Y)^t^G(Y)^g^A(Y)^a^T(Y)^g^5(Y)^a	18	Figure 1<br>Figure S2
AmNA[+1/+18]	A(Y)^a^T(Y)^g^G(Y)^t^A(Y)^t^5(Y)^c^A(Y)^t^A(Y)^c^5(Y)^c^5(Y)^a	18	Figure 1<br>Figure S2
AmNA[+13/+30]	T(Y)^a^A(Y)^a^T(Y)^a^T(Y)^t^A(Y)^c^5(Y)^a^A(Y)^a^T(Y)^g^G(Y)^t	18	Figure 1<br>Figure S2
AmNA[+15/+32]	A(Y)^g^T(Y)^a^A(Y)^a^T(Y)^a^T(Y)^t^A(Y)^c^5(Y)^a^A(Y)^a^T(Y)^g	18	Figure 1<br>Figure S2
AmNA[+35/-2]	A(Y)^c^5(Y)^c^A(Y)^t^5(Y)^t^A(Y)^g^A(Y)^t^5(Y)^a^5(Y)^a^5(Y)^t	18	Figure 1<br>Figure S2
AmNA[+27/+44]	A(Y)^g^A(Y)^t^5(Y)^a^5(Y)^a^5(Y)^t^5(Y)^t^A(Y)^g^T(Y)^a^A(Y)^a	18	Figures 1&2<br>Figure S2
AmNA[+23/+40]	5(Y)^a^5(Y)^a^5(Y)^t^5(Y)^t^A(Y)^g^T(Y)^a^A(Y)^a^T(Y)^a^T(Y)^t	18	Figures 1&2

			Figure S2
AmNA[-44/-27]	A(Y)^a^A(Y)^c^G(Y)^g^A(Y)^a^A(Y)^t^T(Y)^g^A(Y)^c^A(Y)^t^T(Y)^t	18	Figure S1
AmNA[-32/-15]	T(Y)^g^5(Y)^a^T(Y)^a^G(Y)^t^A(Y)^g^A(Y)^a^A(Y)^a^A(Y)^c^G(Y)^g	18	Figure S1
AmNA[-17/-34]	G(Y)^c^A(Y)^t^A(Y)^a^G(Y)^a^G(Y)^t^A(Y)^a^T(Y)^a^5(Y)^a^T(Y)^t	18	Figure S1
AmNA[-8/+10]	5(Y)^c^A(Y)^t^A(Y)^c^5(Y)^c^5(Y)^a^5(Y)^t^G(Y)^g^T(Y)^c^5(Y)^a	18	Figure S1
AmNA[-5/-22]	T(Y)^a^5(Y)^a^T(Y)^t^5(Y)^t^A(Y)^c^5(Y)^t^G(Y)^a^A(Y)^t^A(Y)^c	18	Figure S1
AmNA[+43/-10]	G(Y)^a^A(Y)^t^A(Y)^c^A(Y)^t^A(Y)^c^5(Y)^t^G(Y)^a^A(Y)^t^A(Y)^c	18	Figure S1
AmNA[+23/+38]	5(Y)^a^5(Y)^t^5(Y)^t^A(Y)^g^T(Y)^a^A(Y)^a^T(Y)^a^T(Y)^t	16	Figure 2
AmNA[+25/+40]	5(Y)^a^5(Y)^a^5(Y)^t^5(Y)^t^A(Y)^g^T(Y)^a^A(Y)^a^T(Y)^a	16	Figure 2
AmNA[+27/+42]	A(Y)^t^5(Y)^a^5(Y)^a^5(Y)^t^5(Y)^t^A(Y)^g^T(Y)^a^A(Y)^a	16	Figure 2
AmNA[+29/+44]	A(Y)^g^A(Y)^t^5(Y)^a^5(Y)^a^5(Y)^t^5(Y)^t^A(Y)^g^T(Y)^a	16	Figure 2
AmNA[+26/+43]	G(Y)^a^T(Y)^c^A(Y)^c^A(Y)^c^T(Y)^c^T(Y)^a^G(Y)^t^A(Y)^a^A(Y)^t	18	Figure 2
AmNA[+27/+44]_1/3	A(Y)^g^a^T(Y)^c^a^5(Y)^a^c^T(Y)^c^t^A(Y)^g^t^A(Y)^a^a	18	Figure 2

AmNA[+25/+44]	A(Y)^g^A(Y)^t^5(Y)^a^5(Y)^a^5(Y)^t^5(Y)^t^A(Y)^g^T(Y)^a^A(Y)^a^T(Y)^a	20	Figure 2
AmNA[+27/+46]	5(Y)^t^A(Y)^g^A(Y)^t^5(Y)^a^5(Y)^a^5(Y)^t^5(Y)^t^A(Y)^g^T(Y)^a^A(Y)^a	20	Figure 2
AmNA[+23/+42]	A(Y)^t^5(Y)^a^5(Y)^a^5(Y)^t^5(Y)^t^A(Y)^g^T(Y)^a^A(Y)^a^T(Y)^a^T(Y)^t	20	Figure 2
AmNA[+21/+40]	5(Y)^a^5(Y)^a^5(Y)^t^5(Y)^t^A(Y)^g^T(Y)^a^A(Y)^a^T(Y)^a^T(Y)^t^A(Y)^c	20	Figure 2
AmNA[+23/+44]	A(Y)^g^A(Y)^t^5(Y)^a^5(Y)^a^5(Y)^t^5(Y)^t^A(Y)^g^T(Y)^a^A(Y)^a^T(Y)^a^T(Y)^t	22	Figure 2
NC1	5(Y)^a^5(Y)^c^T(Y)^a^T(Y)^c^G(Y)^t^A(Y)^a^A(Y)^c^A(Y)^c^T(Y)^c	18	Figure S3
NC2	5(Y)^c^A(Y)^c^5(Y)^t^A(Y)^g^5(Y)^a^A(Y)^t^A(Y)^t^5(Y)^a^5(Y)^t	18	Figure S3
NC3	5(Y)^g^5(Y)^c^A(Y)^c^A(Y)^a^5(Y)^t^A(Y)^t^5(Y)^a^5(Y)^t^A(Y)^t	18	Figure S3
NC4	5(Y)^a^5(Y)^g^A(Y)^a^A(Y)^t^5(Y)^t^A(Y)^t^5(Y)^c^5(Y)^c^A(Y)^t	18	Figure S3

The names, sequences, and base numbers of each oligonucleotide were shown. Each oligonucleotide was used in Figure 1, Figure 2, or Figures S1, 2, and 3.

5(Y)=AmNA_mC, N(Y)=AmNA, Lower case=DNA, ^Phosphorothioated.

Table S2. Higher-order structure prediction of REST_SSO by in silico analysis

Name	Oligonucleotide sequence (5'→ 3')	Intramolecular FE [kcal/mol]	Intermolecular FE [kcal/mol]
AmNA[+23/+42]	ATCACACTCTAGTAAATATT	−0.10	−1.05
AmNA[+21/+40]	CACACTCTAGTAAATATTAC	−0.23	−1.65
AmNA[+23/+38]	CACTCTAGTAAATATT	−0.03	−1.14
AmNA[+25/+40]	CACACTCTAGTAAATA	−0.09	−1.16
AmNA[+27/+46]	CTAGATCACACTCTAGTAAA	−3.49	−7.64
AmNA[+25/+44]	AGATCACACTCTAGTAAATA	−0.38	−2.13
AmNA[+27/+42]	ATCACACTCTAGTAAA	−0.09	−1.15
AmNA[+29/+44]	AGATCACACTCTAGTA	−0.37	−2.18
AmNA[+23/+44]	AGATCACACTCTAGTAAATATT	−0.38	−2.38
AmNA[+26/+43]	GATCACACTCTAGTAAAT	−0.14	−2.57
AmNA[-24/-7]	CAATGGAATGCATAGTAG	−0.61	−5.42
AmNA[-18/-1]	CTGGTCCAATGGAATGCA	−1.03	−9.17
AmNA[+1/+18]	AATGGTATCCATACCCCA	−2.48	−8.39
AmNA[+13/+30]	TAAATATTACCAAATGGT	−1.34	−5.61
AmNA[+15/+32]	AGTAAATATTACCAAATG	−0.38	−2.07
AmNA[+23/+40]	CACACTCTAGTAAATATT	−0.10	−1.09
AmNA[+27/+44]	AGATCACACTCTAGTAAA	−0.37	−2.08
AmNA[+35/-2]	ACCCATCTAGATCACACT	−0.01	−6.00

Free energy (FE) of the thermodynamic ensemble was calculated in a higher-order structure based on intramolecular or intermolecular interactions. Each sequence is shown without distinct AmNA and DNA. Refer to Table S1 for detailed sequences.

Table S3. Microarray analysis (mRNAs)

Gene Symbol	AmNA[+21/+40]		AmNA[+23/+44]		ChIP-Atlas (REST)
	Ratio	p-value	Ratio	p-value	Threshold (>500)
BPIFA4P	0.50	0.0489	0.90	0.0899	RE1
MIR762HG	2.46	<0.0001	2.08	<0.0001	(-)
KCNB1	0.48	0.0126	0.54	0.0695	RE1
ANKRD34C-AS1	0.38	0.0060	0.67	0.0452	RE1
XKR7	0.31	0.0005	0.74	0.0630	RE1
ZKSCAN8P1	3.02	0.0004	1.54	0.0199	(-)
SVOP	0.42	0.0004	0.83	0.0591	RE1
ACTL6B	0.45	0.0046	0.83	0.0138	RE1
KIAA0408	0.49	0.0078	0.84	0.1322	(-)
KEL	0.43	0.0311	0.77	0.4404	RE1
RRH	2.20	0.0009	1.29	0.0199	(-)
LAT2	4.98	0.0001	0.94	0.4110	(-)
CDO1	0.18	0.0334	0.33	0.1141	RE1
GABRD	0.45	0.0128	0.94	0.7998	RE1
DISP2	0.38	0.0068	0.86	0.0572	RE1
LOC107985773	0.48	0.0246	0.74	0.2462	(-)
SYN1	0.37	0.0009	0.75	0.0054	RE1
MIR7-3HG	0.21	0.0008	0.69	0.0201	RE1

CABP1	0.49	0.0132	0.95	0.7402	RE1
HRH3	0.47	0.0061	0.90	0.3754	RE1
SRRM3	0.44	0.0001	0.78	0.0046	RE1
UNC79	0.44	0.0011	0.91	0.2910	RE1
INSM2	0.40	0.0020	1.04	0.6396	RE1
SEZ6	0.29	0.0002	0.82	0.0298	RE1
CHGB	0.45	0.0008	0.81	0.0423	RE1
CPNE9	0.30	0.0031	0.74	0.1077	RE1
TMEM145	0.49	0.0036	0.82	0.1217	RE1
PSORS1C2	2.06	0.0477	1.50	0.2631	RE1
DNAI7	4.17	0.0100	2.44	0.0163	(-)
SPTBN4	0.41	0.0129	0.83	0.3644	RE1
C17orf98	4.98	0.0082	1.15	0.4590	(-)
CPLX2	0.29	0.0002	0.76	0.0367	RE1
KCNS2	0.33	0.0145	0.58	0.1056	RE1
UNC80	0.38	0.0331	0.62	0.1728	RE1
CDKN1A	2.97	0.0018	1.14	0.3836	RE1
GNAO1	0.32	0.0360	0.63	0.0109	RE1
SLITRK1	0.43	0.0037	0.88	0.2143	RE1
CACNA1B	0.42	0.0032	0.85	0.2236	RE1
SNTG1	0.49	0.0234	0.80	0.3731	RE1

KDSR	2.07	0.0047	1.18	0.0574	(-)
CHRNB2	0.25	0.0004	0.72	0.0114	RE1
TRIM67	0.32	0.0090	0.74	0.0243	RE1
KCNC1	0.38	0.0031	0.80	0.0741	RE1
HPCA	0.49	0.0132	0.81	0.1718	RE1
SCRT2	0.44	0.0003	0.88	0.1182	RE1
FCER1G	0.48	0.0277	0.64	0.1318	RE1
CHGA	0.43	0.0010	0.85	0.0688	RE1
RAB3C	0.44	0.0210	0.93	0.7265	RE1
SNAP25	0.37	0.0001	0.77	0.0169	RE1
RAB44	6.08	0.0007	1.01	0.9091	(-)
ATP1A1	0.33	0.0133	0.54	0.2141	RE1
MMP24	0.44	<0.0001	0.84	0.0407	RE1
LRIT3	15.97	0.0029	2.94	0.0133	(-)
SCRT1	0.42	0.0045	0.85	0.0290	RE1
GJA4	2.01	0.1412	3.11	0.0498	(-)
CALR3	1.18	0.3187	2.38	0.0006	(-)
PPFIA4	0.90	0.5339	0.48	0.0195	RE1
PRELID2	0.85	0.4259	0.43	0.0227	RE1
REST	1.14	0.1413	1.27	0.0855	RE1
SRRM4	0.77	0.0153	0.87	0.1940	RE1

Ratio compared with the negative control (NC) was shown based on the results of data analysis with changes of over 2-fold.

REST and SRRM4 were added to the list as reference RE1 genes.

Table S4. Microarray analysis (miRNAs)

Systematic name	AmNA[+21/+40]		AmNA[+23/+44]		AmNA7168	
	Ratio	p-value	Ratio	p-value	Ratio	p-value
hsa-miR-151a-3p	0.437	0.0407	0.612	0.1078	0.518	0.0616
hsa-miR-18b-5p	0.488	0.0444	0.598	0.0637	0.562	0.0774
hsa-miR-23b-3p	0.560	0.0566	0.640	0.1104	0.490	0.0286
hsa-miR-4281	1.917	0.0006	2.024	0.0006	1.982	0.0007
hsa-miR-4459	2.148	0.0148	2.225	0.0193	3.077	0.0012
hsa-miR-4465	1.404	0.0867	1.660	0.0535	2.020	0.0161
hsa-miR-4516	2.323	0.0026	2.793	0.0003	2.588	0.0010
hsa-miR-5703	3.879	0.0119	4.495	0.0069	5.545	0.0003
hsa-miR-5787	2.780	0.0007	3.225	0.0004	4.021	0.0000
hsa-miR-6087	1.704	0.0166	1.746	0.0232	2.040	0.0039
hsa-miR-6090	2.359	0.0000	2.528	0.0000	2.745	0.0001
hsa-miR-630	3.733	0.0097	4.215	0.0063	5.315	0.0004
hsa-miR-642a-3p	1.598	0.0128	1.795	0.0150	2.526	0.0005
hsa-miR-6858-3p	0.499	0.1212	0.541	0.2340	0.469	0.0474
hsa-miR-7107-5p	1.954	0.0053	1.794	0.0158	2.024	0.0011
hsa-miR-7150	3.624	0.0042	3.804	0.0031	5.227	0.0000
hsa-miR-762	2.107	0.0459	2.819	0.0120	2.844	0.0124
hsa-miR-8063	1.761	0.0012	1.943	0.0066	2.532	0.0002

hsa-miR-9-5p	0.430	0.0204	0.552	0.0505	0.559	0.0487

Ratio was analysed compared with the non-treatment control.

Figure S1.

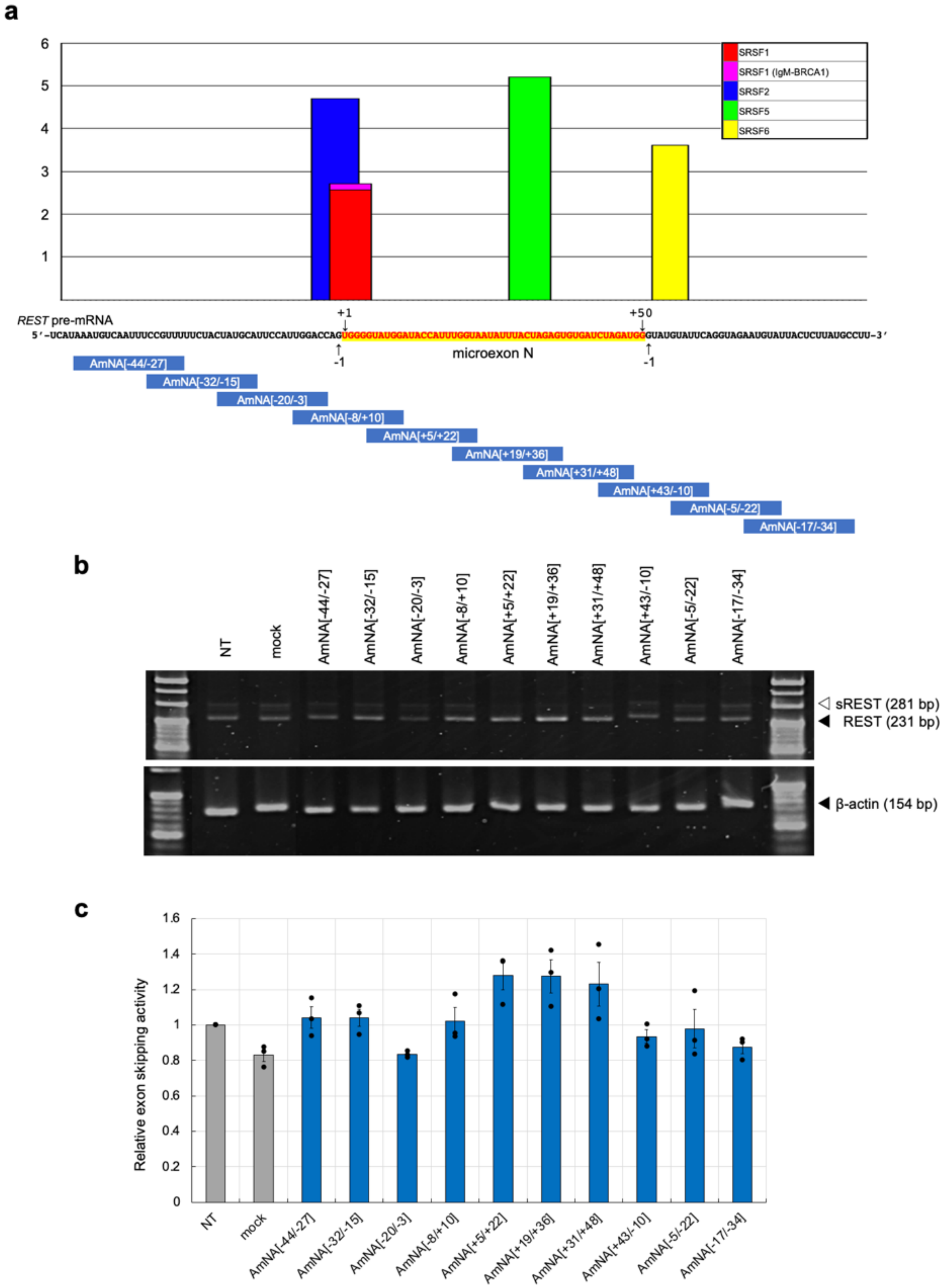
a
SRSF1
SRSF1 (IgM-BRCA1)
SRSF2
SRSF5
SRSF6
REST pre-mRNA
+1
+50
-1
microexon N
AmNA[-44/-27]
AmNA[-32/-15]
AmNA[-20/-3]
AmNA[-8/+10]
AmNA[+5/+22]
AmNA[+19/+36]
AmNA[+31/+48]
AmNA[+43/-10]
AmNA[-5/-22]
AmNA[-17/-34]
b
NT
mock
sREST (281 bp)
REST (231 bp)
β-actin (154 bp)
c
Relative exon skipping activity
NT
mock

Figure S1: Skipping of *REST* microexon N by REST_SSOs in STC-1 cells. a, Schematic of microexon N (highlighted in yellow) in the *REST* pre-mRNA. Oligonucleotides were designed around microexon N and are shown under *REST* pre-mRNA. The numbers in parenthesis indicate where the 5′ and 3′ ends are located on the pre-mRNA, with negative numbers indicating introns and positive numbers indicating exons. The diagram above the *REST* pre-mRNA shows the prediction of the SRSF-binding positions and ESE homology score on the vertical axis based on ESEfinder3.0. **b,** Screening of REST SSOs using SCLC cells (STC-1). STC-1 cells were transfected with each oligonucleotide (final concentration, 10 nM) via electroporation. After 24 h of transfection, RNA was extracted, and the REST band was analysed via agarose gel electrophoresis. **c,** Exon skipping activity for each oligonucleotide was evaluated compared with that of NT, which was set as 1 (n = 3, mean ± standard error of mean). Exon skipping activity was defined as the intensity of the REST band relative to the sum of the intensities of the REST and sREST bands. NT: non-treatment; mock: lipofection without SSO.

Figure S2

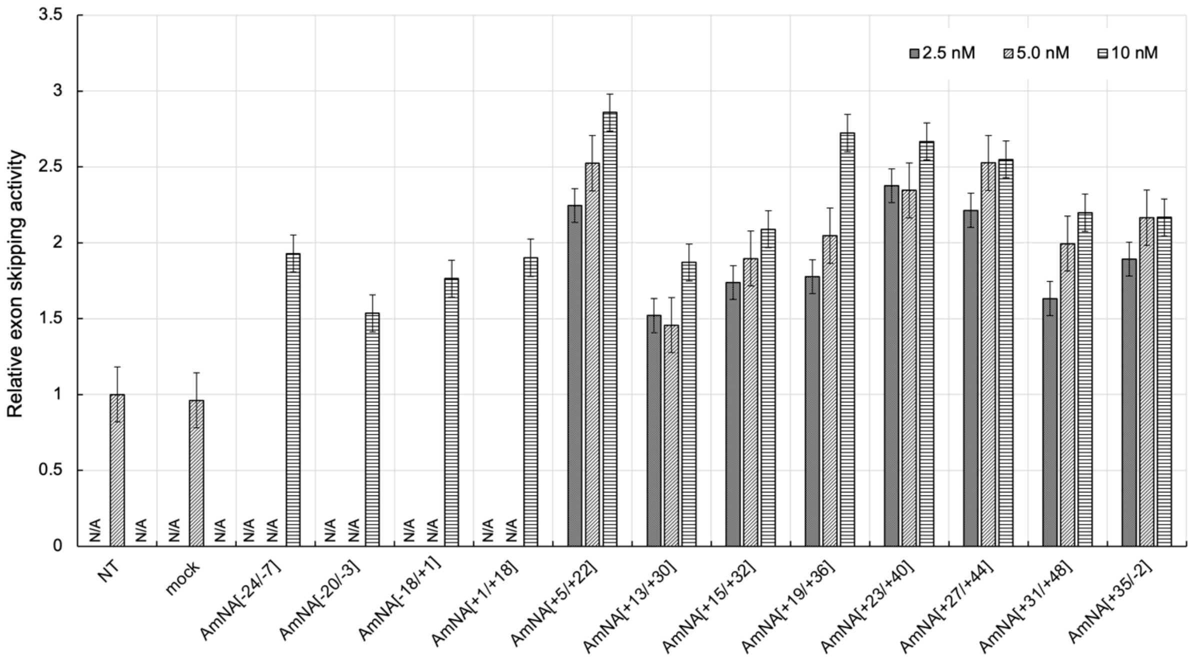

Figure S2: Analysis of exon skipping by REST SSOs in VCaP cells. The PCa cell line VCaP was transfected with various amounts of oligonucleotides, and total RNA was extracted after 48 h. RT-PCR was performed to assess *REST* splicing, and exon skipping activity was analysed based on band intensities on polyacrylamide gels. Exon skipping activity is shown compared with that of NT, which was set as 1 (n = 3, mean ± standard error of mean). NT: non-treatment; mock: lipofection without SSO. AmNA[-24/-7], AmNA[-20/-3], AmNA[-18/+1], and AmNA[+1/+18] were used at a concentration of 10 nM, whereas NT and mock were used at 5.0 nM. NA; not available.

Figure S3

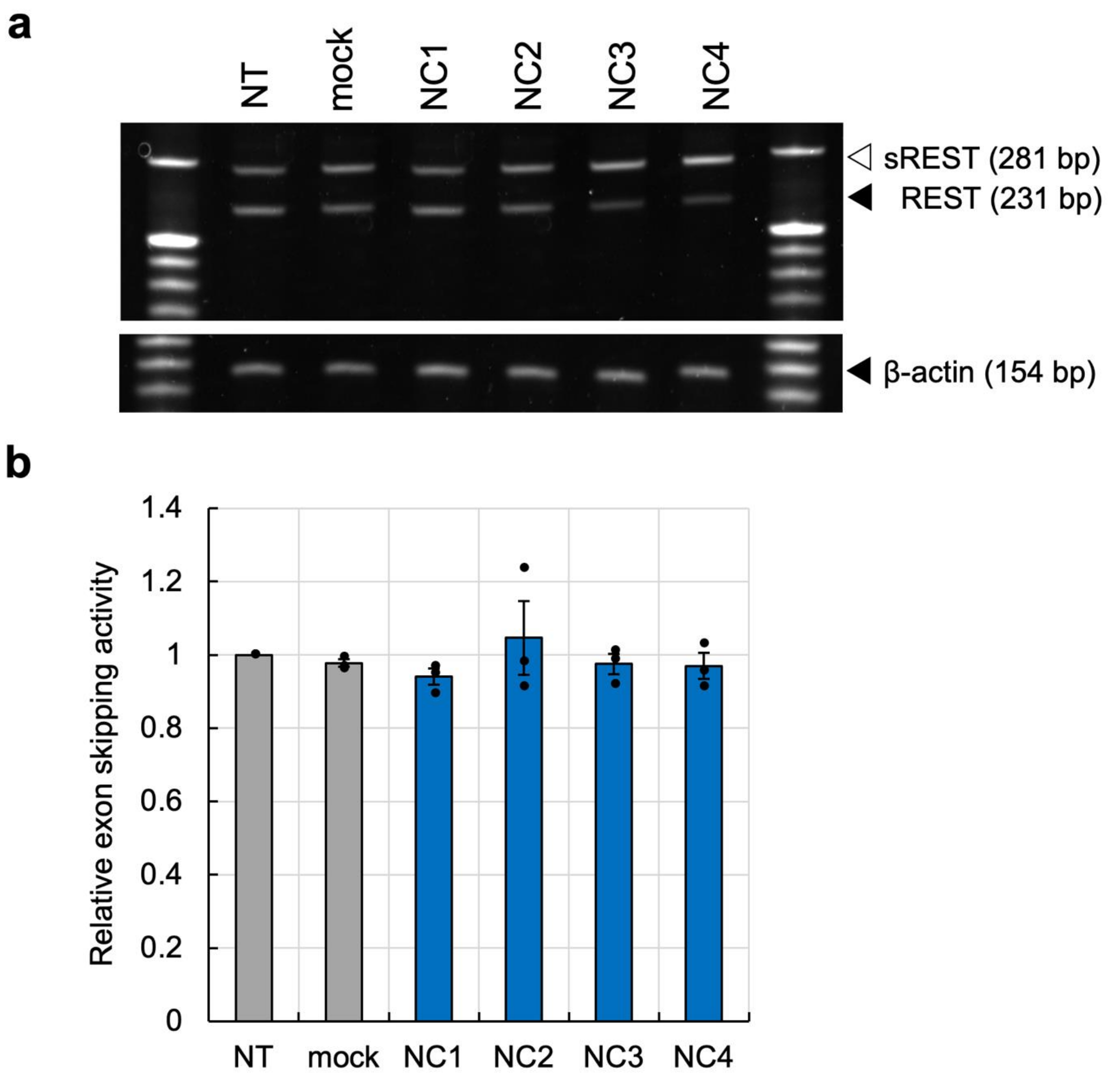

Figure S3: Assessment of negative control oligonucleotides using the PCa cell line VCaP. **a,** VCaP cells were transfected with each oligonucleotide (NC1-4) (final concentration: 10 nM) using Lipofectamine, and total RNA was extracted after 24 h. RT-PCR was performed and REST (231 bp) and sREST (281 bp) were separated via polyacrylamide gel electrophoresis. β-actin (154 bp) was used as the internal standard. **b,** Exon skipping activity for each oligonucleotide was evaluated compared with that of the NT control, which was set as 1 (n = 3, mean ± standard error of mean). Exon skipping activity was defined as the intensity of the REST band relative to the sum of the intensities of the REST and sREST bands. NT: non-treatment; mock: lipofection without SSO.

Figure S4

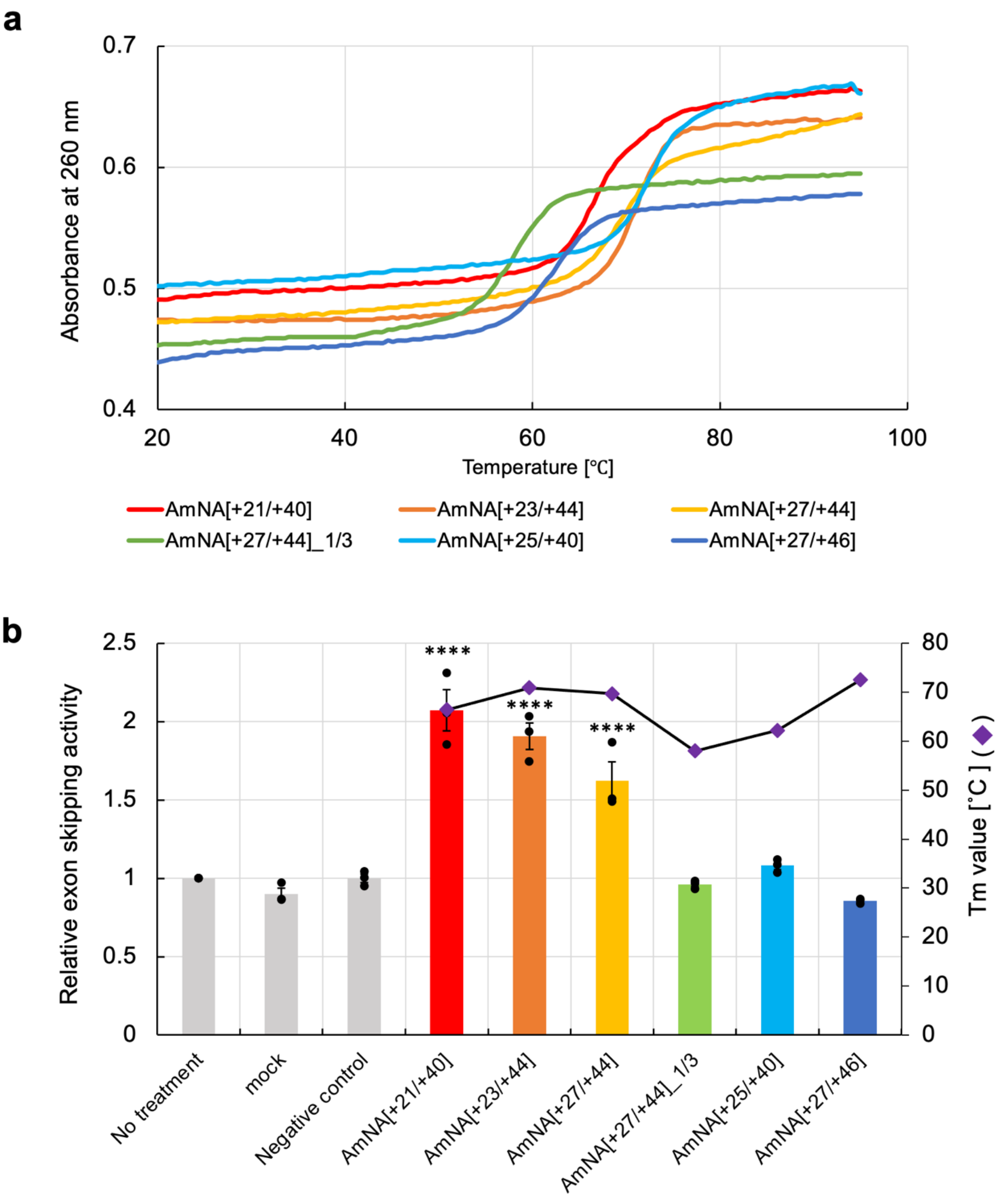

Figure S4: *T*m evaluation of each oligonucleotide. a, *T*m melting curves for each oligonucleotide. **b,** Each oligonucleotide was analysed based on the melting temperature curve. Exon skipping activity is shown in the bar graph, and *T*m indicated on the right is shown as a diamond above the bar graph. mock: lipofection without SSO. Statistical significance is expressed in comparison with values for no treatment and was analysed using one-way analysis of variance (ANOVA) followed by Dunnett's t-test. ****P < 0.0001.

Figure S5

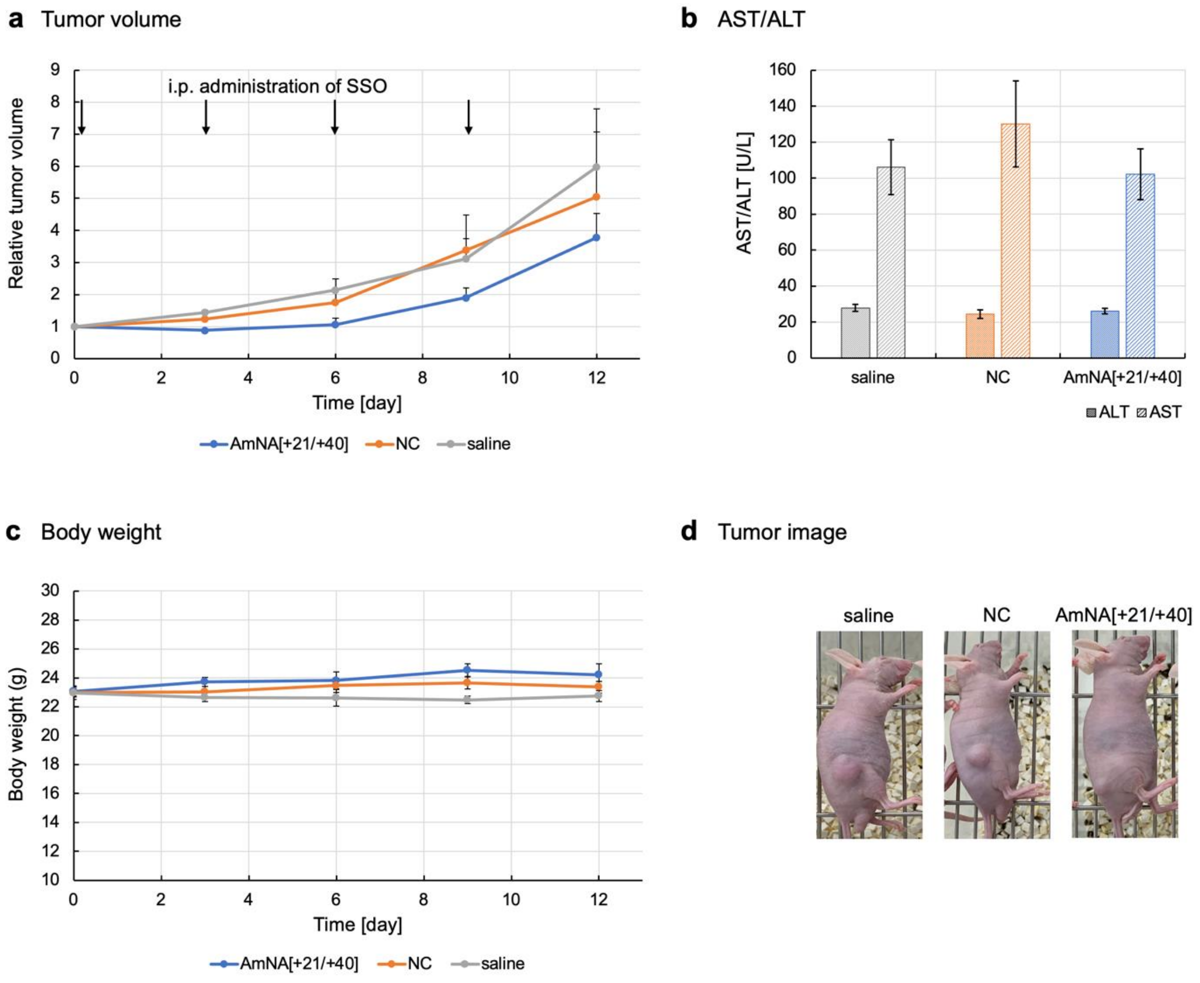

Figure S5: Antitumour effects after AmNA[+21/+40] administration in xenograft mice bearing tumours derived from SCLC cell line N417. a, Antitumour effects upon AmNA[+21/+40] administration in SCLC tumour-bearing xenograft mice. N417 cells (5.0 $\times 10^5$) were subcutaneously implanted into 8-week-old BALB/c Slc-nu/nu mice (n = 5); after 7 days, saline or oligonucleotides (AmNA[+21/+40] or NC) were intraperitoneally administered at 10 mg/kg every 3 days. Tumour volume was calculated based on the long and short diameters. **b,** AST/ALT tests using blood samples. Blood samples were collected on day 12 and used for the ALT/AST assay. **c**, Body weight was measured every 3 days. **d,** Images of tumours observed for mice from different groups. NC; negative control.

Figure S6

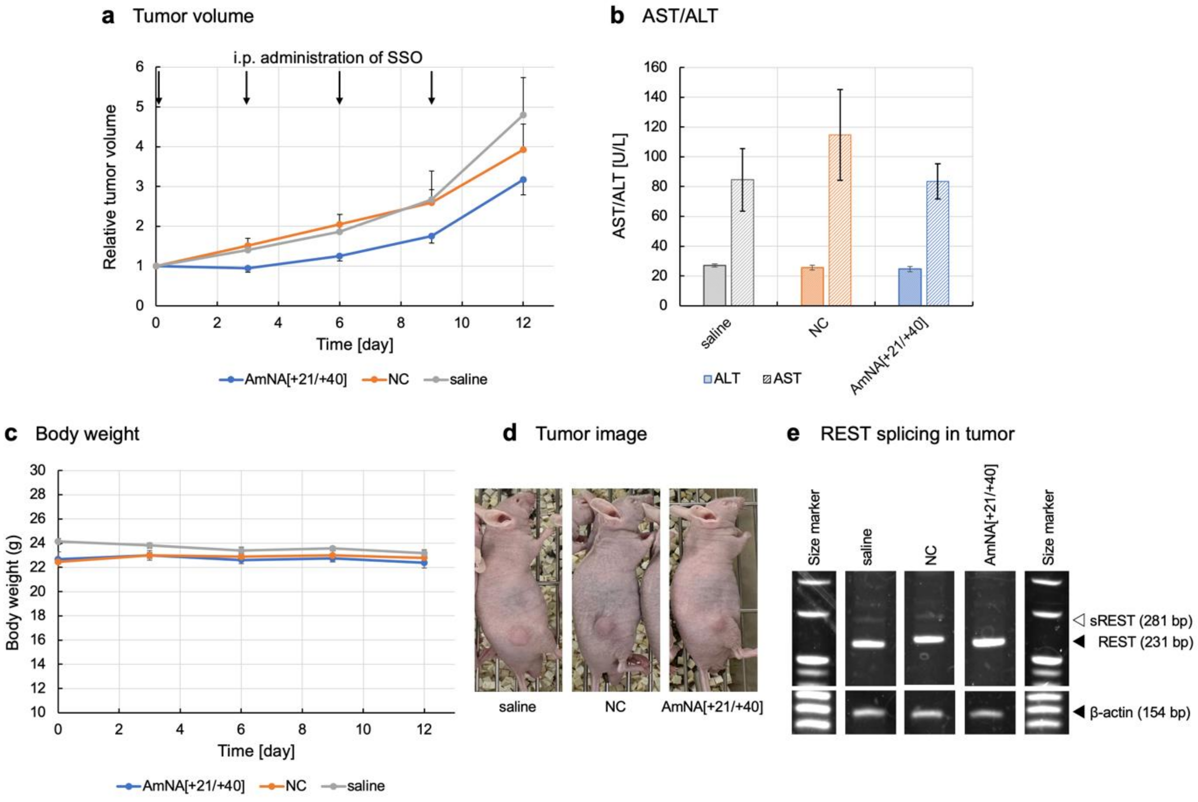

Figure S6: Antitumour effects upon AmNA[+21/+40] administration in xenograft mice bearing tumours derived from the PCa cell line 22Rv1. a, Antitumour effects upon AmNA[+21/+40] administration in PCa tumour-bearing xenograft mice. 22Rv1 cells (5.0×10^5) were subcutaneously implanted into 8-week-old BALB/c Slc-nu/nu mice (n = 5); after 7 days, saline or oligonucleotides (AmNA[+21/+40] or NC) were intraperitoneally administered at a dose of 10 mg/kg every 3 days. Tumour volume was calculated. **b,** AST/ALT test using blood samples on day 12. **c**, Body weight was measured every 3 days. **d,** Images of tumours observed for mice from different groups. **e,** *REST* splicing in the tumour was analysed. NC; negative control.

Figure S7

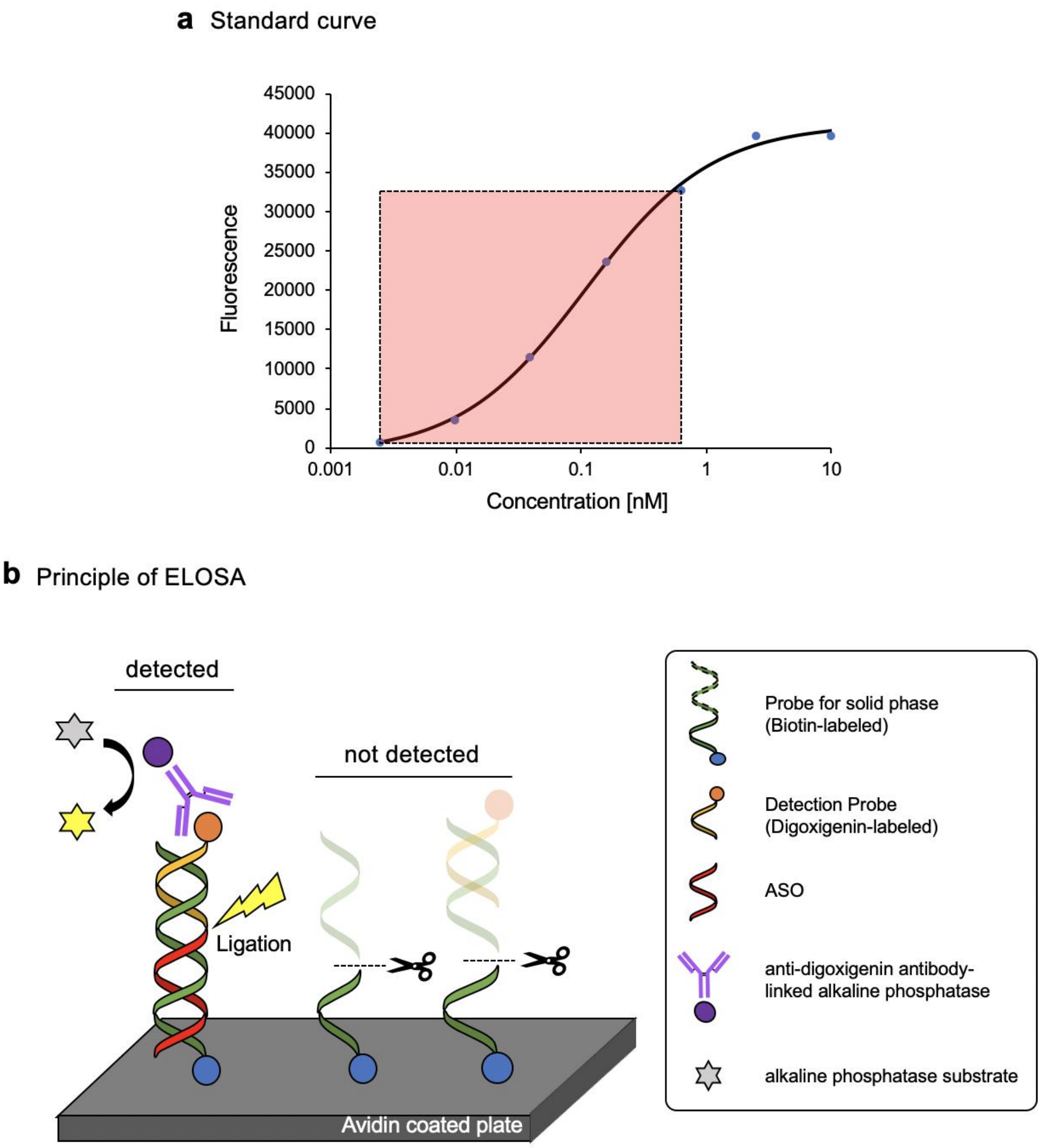

Figure S7: Principle of oligonucleotide quantification by enzyme-linked oligosorbent assay (ELOSA). a, Standard curve obtained using known amounts of AmNA[+21/+40]. **b,** Principle of ELOSA.

Figure S8.

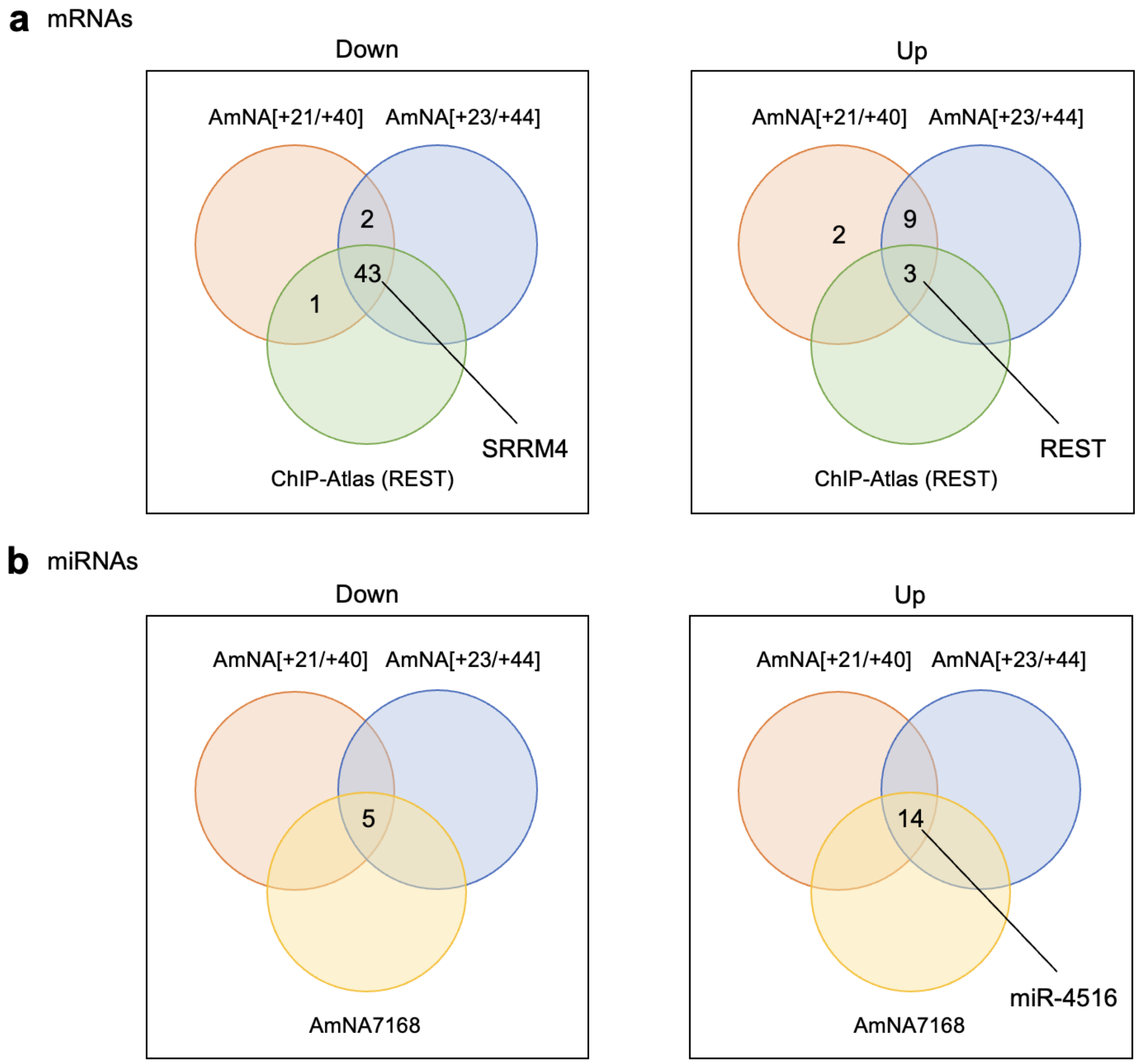

Figure S8: Microarray analysis in 22Rv1 cell transfected with AmNA[+21/+40] or AmNA[+23/+44]. The number of genes whose expression levels changed by more than 2-fold has been shown in the Venn diagram. **a,** mRNA **b,** miRNA
